# Fish connectivity mapping: linking chemical stressors by their mechanisms of action-driven transcriptomic profiles

**DOI:** 10.1186/s12864-016-2406-y

**Published:** 2016-01-28

**Authors:** Rong-Lin Wang, Adam D. Biales, Natalia Garcia-Reyero, Edward J. Perkins, Daniel L. Villeneuve, Gerald T. Ankley, David C. Bencic

**Affiliations:** Exposure Methods & Measurements Division, National Exposure Research Laboratory, US Environmental Protection Agency, 26 W Martin Luther King Dr., MS 587, Cincinnati, OH 45268 USA; Environmental Laboratory, US Army Engineer Research and Development Center, US Army Corps of Engineers, 3909 Halls Ferry Rd, Vicksburg, MS 39180 USA; Mid-Continent Ecology Division, National Health and Environmental Effects Research Laboratory, US Environmental Protection Agency, 6201 Congdon Boulevard, Duluth, MN 55804 USA

**Keywords:** Fish, Gene expression profiles, Connectivity mapping

## Abstract

**Background:**

A very large and rapidly growing collection of transcriptomic profiles in public repositories is potentially of great value to developing data-driven bioinformatics applications for toxicology/ecotoxicology. Modeled on human connectivity mapping (Cmap) in biomedical research, this study was undertaken to investigate the utility of an analogous Cmap approach in ecotoxicology. Over 3500 zebrafish (*Danio rerio*) and fathead minnow (*Pimephales promelas*) transcriptomic profiles, each associated with one of several dozen chemical treatment conditions, were compiled into three distinct collections of rank-ordered gene lists (ROGLs) by species and microarray platforms. Individual query signatures, each consisting of multiple gene probes differentially expressed in a chemical condition, were used to interrogate the reference ROGLs.

**Results:**

Informative connections were established at high success rates within species when, as defined by their mechanisms of action (MOAs), both query signatures and ROGLs were associated with the same or similar chemicals. Thus, a simple query signature functioned effectively as an exposure biomarker without need for a time-consuming process of development and validation. More importantly, a large reference database of ROGLs also enabled a query signature to cross-interrogate other chemical conditions with overlapping MOAs, leading to novel groupings and subgroupings of seemingly unrelated chemicals at a finer resolution. This approach confirmed the identities of several estrogenic chemicals, as well as a polycyclic aromatic hydrocarbon and a neuro-toxin, in the largely uncharacterized water samples near several waste water treatment plants, and thus demonstrates its future potential utility in real world applications.

**Conclusions:**

The power of Cmap should grow as chemical coverages of ROGLs increase, making it a framework easily scalable in the future. The feasibility of toxicity extrapolation across fish species using Cmap needs more study, however, as more gene expression profiles linked to chemical conditions common to multiple fish species are needed.

**Electronic supplementary material:**

The online version of this article (doi:10.1186/s12864-016-2406-y) contains supplementary material, which is available to authorized users.

## Background

There are an estimated 80,000 chemicals in legal use today, with hundreds more added to the inventory each year [1]. A current lack of toxicological information for most of them poses a serious challenge to safeguarding human health and the environment from potentially harmful exposures. Given that traditional toxicity testing of chemicals based on whole animals is resource-intensive, time-consuming, and at times challenging in terms of cross-species and –chemical extrapolation, a general consensus has emerged across the environmental science community that alternative practices are needed for evaluation and management of chemical inventories. In recognition of rapid scientific advancements in “-omics” technologies, robotics, computational chemistry, systems biology, and high performance computing, the US National Academy of Sciences has put forth several reports since the turn of the century [2–5] recommending broader utilization of *in vitro, in silico*, and short term *in vivo* assays with a greater focus on mechanistic pathways in testing of chemicals and assessing their toxicological risks.

Concurrent with this emerging paradigm of toxicology is the ongoing “big data” revolution across scientific disciplines and technological fields. Accelerated by technical advances, there has been an exponential growth in data generation. In biology, for example, the number of DNA sequence bases in GenBank has increased nearly 300,000 fold over the last 30 years, from 0.7 megabases in 1982 to 187 gigabases in 2015. As of May, 2015, the NCBI GEO (National Center for Biotechnology Information Gene Expression Omnibus) hosted well over 1.4 million gene expression profiles (GEPs), including ~20,000 for fish, ~70,000 for rats, and ~700,000 for humans. Each GEP represents the collective expression states of all genes, as measured by a given microarray, for a sample under study. Many of these GEPs are linked to chemical treatment or other biological conditions of potential relevance to toxicology. These abundant transcriptomic data contain a wealth of information and present opportunities for toxicologists to explore computational assessment of chemical toxicity by a data-driven approach. In contrast to individual studies with a narrowly defined scope and limited data, substantial novel insights may be gained from data mining across a large number of independent studies conducted within the same species or even across species. Yet, to date, there has been little research effort in this area in the field of toxicology.

Connectivity mapping (Cmap) represents an *in silico* and data-driven approach with potentially broad applications in biomonitoring, chemical exposure assessment, toxicity evaluation and extrapolation across species, and grouping of chemicals. Originally proposed for human biomedical research [6], Cmap connects chemicals and disease based on similarities in transcriptomic profiles, driven largely by the underlying mechanisms of action (MOA). Such similarities are revealed by interrogating a database of rank-ordered gene lists (ROGLs) with a query signature. The ROGLs are generated individually from GEPs of treated samples relative to those of the corresponding controls, based on gene probes sorted by their logarithmic fold-changes (LogFCs), and are inclusive of all gene probes on a given microarray. A query signature, on the other hand, contains only a small number of gene probes differentially expressed under a chemical or biological condition of interest. A non-random distribution of gene probes from a query signature on ROGLs suggests a similarity in their transcriptomic profiles, and therefore, a connectivity of the underlying chemical or biological conditions. This Cmap determination of the chemical identity associated with a biological sample is thus analogous to forensic database searches by human or DNA fingerprints. Since its inception, Cmap has made a significant impact on drug discovery research and development [7], and is now becoming part of much more ambitious public effort of profiling cell signatures [8]. A similar data-driven approach for pharmaceutical research has also been launched using commercial platforms [9]. For toxicogenomic research with chemicals, the principle of Cmap is equally applicable: those toxicants sharing the same or similar MOAs should yield comparable transcriptomic profiles, and connect with one another [10–12].

Compared to other computational approaches with similar toxicological applications, Cmap has several advantages. First, it is algorithmically simple. Connectivity between two chemical conditions is established simply based on a non-random distribution of multiple gene probes on ROGLs. Second, the information in each GEP is fully preserved, as there is no statistical filtering with regard to generating ROGLs: the entire set of gene probes in the GEP of a treated sample is ranked from top to bottom by their LogFCs relative to the corresponding control(s). Third, Cmap is easily scalable. As more GEPs become available, ROGLs can be simply added individually to an existing reference database without any restructuring or reanalysis. With more ROGLs in the database, there is a greater coverage of chemicals and biological conditions, so the power and applicability of Cmap also increase. Lastly, Cmap is cost-effective and user-flexible. As publicly-available GEPs continue to grow, they can be included in a database to expand chemical coverage. An end user can derive a query signature for a sample/condition of interest from a variety of sources such as literature, microarrays, RT-PCRs (reverse transcription polymerase chain reaction), or a public “-omics” data repository.

However, adopting a big data approach such as Cmap for toxicogenomics applications faces many challenges. Unlike human Cmap where GEPs originated from relatively homogeneous cell cultures from a single species, the development of Cmap for fish must take into account data heterogeneity as a result of differences in experimental designs and lab practices among independent studies. Technical factors such as choice of expression profiling platforms (e.g., microarrays vs RNA sequencing) and evolving designs within a platform (e.g., microarrays) may further complicate data integration. Lastly and perhaps most challenging of all, conducting interspecific Cmap introduces further evolutionary complexity.

Interspecific Cmap requires a signature derived from one species to interrogate ROGLs of one or more other species. If successful, this allows the broadest possible inclusion of GEPs available in public repositories, and can inform toxicity extrapolation from model to non-model species. Interspecific Cmap depends on the conservation of both genomes and transcriptomes. In the presence of such conservation, genome annotations become the most limiting factor for interspecific Cmap. Given these considerations, zebrafish and fathead minnow appear to be the two very suitable fish species for a preliminary test of Cmap across species. As a common biological model with extensive genome-level knowledge, zebrafish is a species of choice for many studies, particularly in developmental biology [13]. As such, zebrafish has the largest number of public GEPs available among all fish species. The fathead minnow, on the other hand, has been the dominant aquatic vertebrate test organism in regulatory toxicity testing for decades [14]. Estimated to have shared a last common ancestor 31 million years ago [15], both the zebrafish and fathead minnow are members of the family Cyprinidae, with well-conserved genomes and transcriptomes [16].

This study was undertaken to explore the applications of Cmap in ecotoxicology. The goals were three-fold. First, Cmap was evaluated for connecting the same chemicals within species. In other words, both a query signature and ROGLs under consideration were selected from a single species and associated with an identical chemical. Second, Cmap was tested on related chemicals of the same MOA class within species, for example, by interrogating ROGLs linked to the natural estrogen 17β-estradiol (E2) with a signature linked to the synthetic estrogen 17α-ethinylestradiol (EE2). If successful, Cmap would not only simplify the development of exposure biomarkers, but also provide an alternative way to characterize the extent of overlap among MOAs of related chemicals and inform as to their relative toxicity. Lastly, based on a small number of chemical conditions shared between species, a preliminary trial of interspecific Cmap was conducted to identify issues critical to a more thorough feasibility study in the future.

## Methods

### Chemicals and field water samples

The data employed in the present study were derived from experiments with a number of chemicals (Table 1). The use or source of these chemicals ranges from pesticides, medicine, industrial chemicals or by products, personal care products, and fossil fuel contaminants. In the context of adverse outcome pathways, these chemicals could be grouped by their molecular initiating events (MIEs) [17]. A MIE is defined here as a molecular interaction between a xenobiotic and a specific biomolecule. Since many of these MIEs involve various receptors and enzymes commonly considered as part of the hypothalamic pituitary gonadal (HPG) axis [18], a large portion of chemicals under this study, for example E2 and EE2, are effectively HPG-active toxicants. Also included, among others, were several pyrethroid insecticides (bifenthrin, permethrin, esfenvalerate, cypermethrin) targeting neuro-transmission, and polycyclic aromatic hydrocarbons (PAHs).

**Table 1:** Chemicals and field mixtures associated with the exposure experiments considered in the current study

Chemicals/field mixtures	Use/source	Putative MIE	References
1,4-dimethoxybenzene (DMB)	Ingredient in consumer products	---	---
2,3,7,8-tetrachlorodibenzo-p-dioxin (TCDD)	Industrial byproduct	aromatic hydrocarbon (Ah) receptor agonist	[48]
2,4-dinitrophenol (DNP)	Antiseptic agent, pesticide, industrial chemical	Uncoupling oxidative phosphorylation	---
benz(a)anthracene (BAA)	Fossil fuels	DNA mutagen and Ah receptor agonist (PAH)	[49]
perfluorinated chemicals (PFC)	Industrial chemical	---	---
tert-Butylhydroquinone (TBHQ)	Ingredient in consumer products	---	---
Phenanthrene (PHE)	Fossil fuels	DNA mutagen and Ah receptor agonist (PAH)	[49]
Pyrene (PYR)	Fossil fuels	DNA mutagen and Ah receptor agonist (PAH)	[49]
decabromodiphenyl ether (BDE)	Flame retardant		---
dibenzothiophene (DBT)	Fossil fuels	DNA mutagen and Ah receptor agonist (PAH)	[49]
17ß-estradiol (E2)	Endogenous estrogen	ER agonist	[18]
Diethylstilbestrol (DES)	medicine	ER agonist	---
ethniyl estradiol (EE2)	medicine	ER agonist	[18]
fadrozol (FAD)	medicine	CYP19 inhibitor	[18]
fipronil (FIP)	insecticide	GABA receptor antagonist	[18]
flutamide (FLU)	medicine	Androgen receptor antagonist	[18]
genistein (GEN)	phytoestrogen	ER and PPAR agonist	[50]
ketochonazole (KET)	medicine	CYP11A/CYP17 inhibitor	[18]
trenbolone (TRB)	Beef production	AR agonist	[18]
trilostane (TRI)	Veterinary medicine	3βHSD inhibitor	[18]
vinclozolin (VIN)	fungicide	AR antagonist (fungicide)	[18]
prochloraz (PRO)	fungicide	CYP17/19 inhibitor	[18]
muscimol (MUS)	research	GABA receptor agonist	[18]
bisphenol A (BPA)	Industrial chemical	ER agonist	[51]
Progesterone (PGST)	Endogenous hormone, medicine	PR agonist	[52]
Dihydrotestosterone (DHT)	Endogenous hormone	AR agonist	[53]
Haloperidol (HAL)	medicine	Dopamine D2 receptor antagonist	[54]
Diazepam (DIA)	medicine	GABA-A receptor modulator	[55]
bifenthrin (BIF)	insecticide	voltage-gated sodium channels disruption	[56]
cypermethrin (CYP)	insecticide	voltage-gated sodium channels disruption	[56]
esfenvalerate (ESF)	Insecticide	voltage-gated sodium channels disruption	[56]
Linuron (LIN)	Herbicide	photosynthesis inhibitor	[57]
Terbufos (TER)	Insecticide	acetylcholine esterase inhibitor	[58]
methylparaben (MPA)	Anti-fungal agent	Uncoupling oxidative phosphorylation	[59]
permethrin (PER)	Insecticide	voltage-gated sodium channels disruption	[56]
Propanil (PPL)	Herbicide	photosynthesis inhibitor	[60]
azinphos-methyl (APM)	Insecticide	acetylcholine esterase inhibitor	[61]
Propranolol (PPLL)	Medicine	β-adrenergic receptor antagonist	[62]
protein kinase C inhibitor 412 (PKC412)	Pharmaceutical compound	tyrosine kinase inhibitor	[63]
Triclocarban (TCC)	Anti-bacterial agent	---	---
Gemfibrozil (GEM)	Medicine	PPAR binding and activation	[64]
cyclotrimethylenetrinitramine (RDX)	Explosive	Neuro-toxin?	---
TNT	Explosive	---	---
Water samples near WWTPs			
Effluent; WWTP, San Diego, California (EFFLa, EFFHa)	Positive for GEM, DIA, E2, PGST	---	[29]
Effluent; WWTP, Los Angeles, California (EFFHb)	Positive for GEM, PGST	---	[29]
Effluent; WWTP, Duluth Minnesota (WLSSD)	Positive for various estrogens, BPA	---	Unpublished observations (Jenna Cavallin US EPA)
Upstream, effluent, downstream, WWTP, Ely Minnesota (ElyUS, ElyEFF, ElyDS)	At least one site positive for various estrogens, BPA, TCC, PAHs, chlorpyrifos	---	[30]
Upstream, effluent, downstream, WWTP, Hutchinson Minnesota (HutUS, HutEFF, HutDS)	At least one site positive for various estrogens, DES, BPA, TCC, PAHs, chlorpyrifos	---	[30]
Upstream, effluent, downstream, WWTP, Rochester Minnesota (RochUS, RochEFF, RochDS)	At least one site positive for various estrogens, BPA, TCC,	---	[30]
storm, stream, waste water; WWTP, Gainesville, Florida (stormH2O, strH2O, wasteH2O)	wasteH2O seasonally positive for BPA, DIA, PFCs	---	[65]

*AR* androgen receptor, *ER* estrogen receptor, *GABA* gamma-aminobutyric acid, *HSD* hydroxysteroid dehydrogenase, *MIE* molecular initiating event, *PAH* polycyclic aromatic hydrocarbons, *PR* progesterone receptor, *PPAR* peroxisome proliferators-activated receptor–α, *WWTP* waste water treatment plant

In addition to experiments with individual chemicals, the data also include exposures to field water conditions (Table 1) sampled near several waste water treatment plants (WWTPs). The water samples were characterized for a limited number of chemicals (generally around 140 analytes) by their original investigators using traditional chemical analysis methods. For the purpose of evaluating Cmap performance, only those chemicals both positively identified therein and also present in the data of current study are listed in the table. Almost all of the water samples had detectable concentrations of estrogens and bisphenol A. Some of them also contained PAHs.

### Microarray data

Microarray data for this investigation came from a series of US Environmental Protection Agency (USEPA) studies with zebrafish and fathead minnow [18] and US Army Corps of Engineers (USACE) studies with fathead minnow, as well as a number of datasets downloaded from NCBI GEO as of August 2014 (Table 2, Additional file 1: Table S1). There were a total of 3516 microarrays associated with 55 experimental conditions, as defined by chemical, dose, tissue type, and exposure duration. This dataset encompassed many independent studies carried out over years, with a wide range of differences in their original project objectives, experimental designs, and chemical treatment conditions and measured effects, using three microarray platforms in zebrafish and fathead minnow. Given the complex and heterogeneous nature of this dataset, it is difficult to describe in general terms all underlying experimental protocols. Instead, only a brief overview of this dataset is outlined below. Greater experimental details about various studies have been previously published [16, 18–20], are available from the summaries of individual data series available at NCBI GEO (Additional file 1: Table S1), or will be published elsewhere.

**Table 2:** A summary of 3516 microarray samples and their chemical treatment conditions

Zebrafish microarray design, sample size, and chemicals	Fathead minnow microarray design, sample size, and chemicals
ZF 21K (Agilent 013223, 15064): USEPA (290)	FHM 15K (Agilent 019597,_036574): USEPA (580)
ethniyl estradiol (**EE2**)^a^	bifenthrin (BIF)^a^
fadrozol (FAD)^a^	cypermethrin (CYP)^a^
fipronil (FIP)^a^	permethrin (PER)^a^
flutamide (**FLU**)^a^	esfenvalerate (ESF)
ketochonazole (KET)^a^	EE2^a^
muscimol (MUS)^a^	Terbufos (TER)^a^
prochloraz (**PRO**)^a^	
trenbolone (TRB)^a^	FHM 15K (Agilent 019597): USACE (1711)
trilostane (TRI)^a^	BPA
vinclozolin (VIN)^a^	FAD^a^
	FLU^a^
ZF 21K (Agilent 013223, 15064): NCBI (154)	Gemfibrozil (GEM)^a^
tert-Butylhydroquinone (TBHQ)^a^	KET^a^
2,3,7,8-tetrachlorodibenzo-p-dioxin (TCDD)^a^	KET_TNT_KET
oxygen (O2)^a^	KET_TNT_TNT
2,4-dinitrophenol (DNP)	PRO
1,4-dimethoxybenzene (DMB)	RDX
azinphos-methyl (APM)^a^	Effluent of Western Lake Superior Sanitary District (WLSSD)
Haloperidol (HAL)	**TRB** ^a^
	TRB_BPA_TRB_BPA
ZF 43K (Agilent 019161): USEPA (24)	TRB_BPA_BPA
FLU^a^	TRB_BPA_TRB
PRO^a^	TRB_EE2_EE2
	TRB_EE2_TRB
ZF 43K (Agilent 019161): NCBI (270)	TRB_EE2_TRB_EE2
benz(a)anthracene (BAA)^a^	TRB_TCC_TCC (triclocarban)
decabromodiphenyl ether (BDE)^a^	TRB_TCC_TRB
bisphenol A (BPA)^a^	TRB_TCC_TRB_TCC
dibenzothiophene (DBT)	TRI^a^
Diazepam (DIA)^a^	VIN^a^
17ß-estradiol (E2)^a^	
**E2** ^a^	FHM 15K (Agilent 019597_036574): NCBI (487)
EE2^a^	Diethylstilbestrol (**DES**)^a^
FLU	Dihydrotestosterone (DHT)
genistein (**GEN**)^a^	E2^a^
Linuron (LIN)^a^	Field exposure (EFFLa, EFFHa^a^; effluent, WWTP San Diego; EFFHb^a^; effluent, WWTP Los Angeles)
methylparaben (MPA)^a^	Field exposure (surface, stream, waste water^a^)
protein kinase C inhibitor 412 (PKC412)^a^	Field exposure (liver: ElyUS^a^, ElyEFF^a^, ElyDS^a^, HutUS, HutEFF^a^, HutDS^a^, RochUS^a^, RochEFF^a^, RochDS^a^))
Propanil (PPL)^a^	Field exposure (ovary: ELYeff, ELYds, HUTeff, HUTds, ROCHds, ROCHeff)
PRO^a^	FLU
Pyrene (PYR)	Linuron
cyclotrimethylenetrinitramine (RDX)^a^	perflorinated chemicals (PFC)
	Phenanthrene (PHE)^a^
	Progesterone (PGST)
	Propranolol (PPLL)
	RDX

Zebrafish: ZF 21K (Agilent 013223, 21495 probes unique; Agilent 015064, 21495 probes), ZF 43K (Agilent 019161, 43603 probes unique). Fathead minnow: FHM 15K (Agilent 019597, 15208 unique probes; Agilent 036574, 15208 of 49849 unique probes). Chemicals with at least one experiment condition (different exposure durations either in single or combinations) with DEGs ≥ 4 are marked by “^a^”, and those shared across platforms or species are highlighted once in bold

### USEPA and USACE studies

A number of experiments were conducted with chemicals targeting HPG-axis [18] and neuro-transmission of zebrafish and fathead minnow. Over the course of these studies, microarray platforms evolved, leading to multiple platforms being used, including Agilent 013223, 015064, 019161 for zebrafish, and Agilent 019597 and 036574 for fathead minnow. Since these platforms had probes duplicated to various extent by design, only unique probes were considered in their cross-mapping. Agilent 013223 and 015064 shared the same 21495 probes but with different layouts (hereafter referred to as ZF 21K). Agilent 019161 had 43603 probes (ZF 43K), but only 37 % (16083) of them could be mapped to Agilent 015064. For fathead minnow, Agilent 019597 had 15208 probes (FHM 15K), which formed a subset of 49849 unique probes found in Agilent 036574 (FHM 60K). The experimental conditions in this study for fathead minnow overlapped considerably between these two platforms. In order to keep their ROGLs as a single collection for maximum comparability, only the 15000 common probes were retained. The entire microarray dataset from these studies is available at NCBI-GEO as the accessions GSE38070, GSE60202, GSE70807, and GSE70936.

### Animal usage

Fish were treated humanely, and all laboratory procedures involving animals were reviewed and approved by the USEPA Animal Care and Use Committee in accordance with Animal Welfare Act regulations and Interagency Research Animal Committee guidelines.

### Zebrafish experiments

Reproductively mature zebrafish (ab wild-type strain, 5–7 months old) were exposed to a continuous flow of sand filtered, UV-sterilized, Lake Superior water (LSW; controls) or test chemicals dissolved in LSW for 24, 48, or 96 h at the USEPA laboratory in Duluth, MN. At the end of each exposure period, fish were anesthetized in a buffered solution of tricaine methanesulfonate (MS-222; Finquel, Argent, Redmond WA, USA) and various tissues were collected and shipped overnight on dry ice to the USEPA laboratory in Cincinnati, OH. Total RNA isolated from selected tissue samples was then sent to Cogenics Corporation, an Agilent certified contract laboratory (Morrisville, North Carolina 27560, USA). Hybridization was conducted using a two-color protocol on ZF 15K and ZF 43K microarrays (Agilent Technologies, Santa Clara, CA, USA), followed by high-resolution scanning and image processing by Agilent Feature Extraction software.

### Fathead minnow experiments

Fish exposures were conducted in the USEPA laboratories in Duluth, MN and Cincinnati, OH; and the USACE laboratories in Vicksburg, MS. For exposures to HPG-active toxicants, reproductively mature fathead minnows (5–7 months old) were tested using LSW as the control and carrier of the test chemicals. All exposures conducted at EPA Duluth laboratories were continuous, flow-through experiments. Representative experimental designs for these experiments are detailed elsewhere [18–20]. Exposures to pyrethroids were conducted in Cincinnati. Fathead minnow fry (48 hours post hatch) and adults (5–7 months old) were exposed to the selected chemicals for 24, 48, or 72 hours in a static or flow-through system. Exposures to TNT, RDX, and mixture of TNT and KET were conducted at USACE Vicksburg laboratories under static renewal conditions using adult fathead minnow (5–7 months old) that will be described elsewhere. At the end of each exposure period, whole fry or tissues from adult fish used for transcriptomic analyses were snap-frozen in liquid nitrogen and stored at −80 ° C until RNA was extracted, using either Qiagen RNeasy mini kits (Qiagen, Valencia, CA, USA) or Tri-Reagent (Sigma, St. Louis, MO, USA). Expression profiling was carried out using a single-color protocol on either a FHM 15K (GEO accession GPL9248, designed by Dr. Nancy Denslow, University of Florida, Gainesville, FL, USA) or a FHM 60K microarray (GPL17098, designed by Dr. Natalia Garcia-Reyero) [21], in the Environmental Laboratory of the US Army Engineer Research and Development Center in Vicksburg, MS (1711 arrays) or in EPA Cincinnati (580 arrays). One hundred to 1000 ng of total RNA was used for all hybridizations. Probe labeling, amplification, and hybridization were performed using Agilent Quick Amp Labeling Kit following the manufacturer’s One-Color Microarray Hybridization Protocol. Microarrays were scanned with a high-resolution scanner and the images were processed with Agilent Feature Extraction software.

### Public data from NCBI GEO

A total of 911 text output files from Agilent Feature Extraction software, representing 33 GEO data series sharing a common microarray platform with those of the USEPA and USACE studies as described above, were assembled and curated (Additional file 1: Table S1). Each sample file was annotated according to chemical exposure, dose, tissue type, and exposure duration.

## Cmap development and analysis

Fish Cmap consists of three components: construction of query signatures from microarray samples of chemical and biological interest, construction of a reference database of ROGLs tagged with chemical or biological conditions, and computational query of the database using the prepared signatures. Each of the three components is outlined below.

### Query signatures

A query signature for a treatment condition contained multiple differentially expressed gene probes (DEGs) relative to an appropriate control. The DEGs were determined by a modified *t*-test implemented in the R package limma [22]. A number of R scripts [23] were developed for this purpose to accommodate different microarray designs such as one-color, two-color direct comparison of treatment and control, two-color with a common reference, and two-color with dye-swaps. Greater detail about DEG determination is available elsewhere [16]. The number of top DEGs selected as a signature and ranked by false discovery rate depended on the types of Cmap query. The Cmap query minimum was lowered from the recommended value of 10 [24], to four DEGs in order to encompass more experimental conditions. On average, though, a signature contained 57 to 88 DEGs for queries made within platforms/species, and 110 to 181 DEGs for Cmap across platforms/species. Intuitively, a larger number of DEGs per signature in the latter case might help to compensate for the possible inconsistencies in GEPs from different microarray platforms within a species, and the possible divergence of chemical MOAs across species. Each DEG in a signature was accompanied by its own value of logarithmic fold-change. In total, 109 unique signatures were prepared for within-platform queries and 61 signatures for queries across platforms/species (Additional file 2: Table S2). These signatures were named after fish sample treatment conditions by the order of chemical, dose, gender, tissue, and exposure duration.

### ROGLs

A ROGL of a treated sample was prepared from appropriate GEPs in several ways depending on study design. For one-color data, gene probe intensities of each treated sample were compared to the corresponding average intensities derived from its specific group of control samples. As a result, each gene probe was given a LogFC of treated over control. Gene probes were then sorted by the absolute values of their LogFC from the smallest to the greatest, and assigned either a positive or negative rank from 1 to N based on the signs of LogFC, with N being number of probes in a given microarray. For two-color direct comparison of a pair of treated over control samples on the same microarray, a ROGL was generated similarly for the treated but within the pair only. For two-color with a common reference design, gene probe intensities of each treated sample were again compared to those averaged over the corresponding reference group. In total, there were 2387 ROGLs in the constructed fish reference database: 386, 203, and 1798 respectively for ZF 21K, ZF 43K, and FHM 15K. For maximum flexibility, the ROGLs from these three platforms were maintained as three distinct collections. Cmap across-platform or across-species was implemented by substituting the probe identifications (IDs) from a source query signature with their equivalent/orthologous probe IDs in its target platform or species.

### Cmap

Interrogation of the fish reference database was conducted using software sscMap [24], containing an algorithm based on the principles of the original Cmap [6]. Basically, Cmap measures the strength of connectivity between a query signature and a ROGL by a connectivity score of their summed ranks. To assess its associated p-value, the default value of 10,000 simulated signatures, each containing the same number of gene probes as the original query signature, are randomly generated from the ROGL. From them connectivity scores are calculated. The p-value for the original query is the proportion of simulated query signatures with their connectivity scores greater than or equal to the observed score. In practice, related ROGLs are typically organized by sscMap into various sets, each defined by some common experimental parameters such as chemical, dosage, and tissue types, and then interrogated by query signatures. Both connectivity score and p-value are slightly modified to account for the variation in the size of individual ROGL sets. With a parameter S denoting the number of ROGL sets contained in a database collection and interrogated by a query signature, the number of false connections was controlled by a critical p-value of 1/S. Each signature was set as “unordered” in the analysis so all up-regulated genes had the same weight of +1, and down-regulated genes had a weight of −1 in contributing to the connectivity score. The highest-scored, statistically significant, and unique pairs of signature-ROGL (excluding those originated from the same experiment) were identified across queries within each platform, and visualized in Cytoscape [25].

To fully evaluate the effectiveness of Cmap, query signatures and ROGLs were arranged into three configurations: within the same platforms and species, across zebrafish platforms, and across species. Because of the differences in coverage of chemicals and their varying transcriptomic impact as measured by the number of DEGs/average LogFC among treatment conditions, queries across platforms and across species were conducted in both directions in order to identify all potential chemical-chemical connections. To enable queries across platforms and species, however, their corresponding probes or orthologs had to be mapped first. This was implemented through several successive steps of ID mapping among ZF 21K, ZF 43K, and FHM 15K microarrays. For example, to identify orthologs between FHM 15K and ZF 21K, the probe sequences from FHM 15K were mapped to their corresponding fathead minnow EST (Expressed Sequence Tag) target sequences (courtesy of Dr. Nancy Denslow, University of Florida) first by TBLASTX. These EST sequences were then mapped to the NCBI nucleotide (NT, as of July, 2013) and protein (NR, as of July 2013) databases by TBLASTX and BLASTX respectively, effectively associating fathead minnow probe IDs to their corresponding NCBI accession IDs. With their greater sequence length, ESTs are presumably more likely than shorter probe sequences to capture orthologs across species. All three rounds of BLAST mapping had a minimum E-value cutoff of E^−06^. These fathead minnow IDs were then joined to a variety of zebrafish accession IDs prepared by the NCBI [26], and finally to Agilent probe annotations [27]. In the end, a total of 9304 probes (43 %) from ZF 21K were linked to 6899 probes (45 %) from FHM 15K through 6573 common Entrez GeneIDs in NCBI. Similarly, 16376 probes (38 %) from ZF 43K were mapped to 9861 probes (65 %) of FHM 15K based on a common set of 10353 Entrez GeneIDs. In addition, 13273 probes (62 %) from ZF 21K mapped to 16083 probes (37 %) of ZF 43K were based entirely on the NCBI “gene2accession” file without using any BLAST programs.

## Results

The performance of Cmap was evaluated by examining the ROGL hits with connectivity scores ranked highest either by individual query signatures or across signatures, based on fish samples profiled on each of the three microarray platforms: ZF 21K, ZF 43K, and FHM 15K. The primary purpose of examining queries individually was to evaluate whether a query signature can indeed connect with its intended ROGL targets when they had commonly associated chemical conditions. This evaluation was carried out both within and across microarray platforms, as well as across fish species. These connections essentially establish the chemical identity of a query signature based on the degree to which chemicals involved have shared MOAs. High-scoring pairs of query signature-ROGL across-queries, on the other hand, could reveal additional novel insights about the MOA similarity of related chemicals. For better clarity in their visualization in a chemical network, each connected pair of chemical conditions was treated as directionless, regardless of which node in the pair represented a signature or a set of ROGLs.

### Validation of Cmap algorithm

Overall Cmap performance was variable, as measured by the percentage of query signatures producing informative connections. An informative connection is arbitrarily defined as a signature and one of its top five ROGL hits sharing the same or similar class of chemicals. Cmap was very effective when both query signatures and ROGLs were from the same microarray platform/species (Table 3). The relatively lower performance in fathead minnow was probably because many treatment conditions had no or very few DEGs (Table 2, Additional file 3: Figure S1). When query signatures and ROGLs were from different platforms and species, the gene probe IDs in a query signature had to be cross-mapped to those in the targeted ROGLs. With the configuration of target probe IDs coupled with source LogFC, an average success rate of 61 % was observed based on a small number of treatment conditions in common across the platforms/species.

**Table 3:** Summary of Cmap by the percentage of query signatures producing informative connections

Microarray platforms	ZF 21K	ZF 43K	FHM 15K
ZF 21K	35/36 (97 %)	11/13 (85 %)	4/8 (50 %)
ZF 43K	6/12 (50 %)	36/36 (100 %)	5/10 (50 %)
FHM 15K	6/12 (50 %)	5/6 (83 %)	35/46 (76 %)

An informative connection was established when a query signature shared the same or similar class of chemicals with at least one of the top five ROGL hits, as ranked by an adjusted connectivity score with a p-value ≤ 1/(S sets of ROGLs). All cross-platform/species Cmap were based on target probe IDs + source gene log-fold changes on shared chemical conditions. Only unique query signatures were considered in calculations

A more detailed examination of connectivity between query signatures and ROGLs provided additional insights into the performance of Cmap. As expected, a query signature almost always connected with ROGLs of its originating treatment condition as the best hit when both came from the same platform (Table 4). In many instances, this connectivity of the same or similar class of chemicals extended across independent experiments, different tissue types, and chemical mixtures as well. For example, a signature from the brain tissue of zebrafish treated with EE2 (EE2_30ngL_M_Brain_48hr) connected with the ROGLs of both EE2_testis and EE2_ovary, overriding the strong impact of tissue types typically observed on fish transcriptomes [28]. Significantly, this strong connectivity among chemical treatment conditions based on their shared MOAs was not limited to HPG-active toxicants. Similarly strong connections were observed in chemicals targeting other biological pathways/processes such as neuro-transmission (BIF_0.15ugL_larvae_48hr), regulation of xenobiotic metabolism (TCDD_2nM_embryo_6h), signal transduction (PKC412_40ugL_fish_6dpf), and photosynthesis (linuron_1.2mgL_embryo_48hr).

**Table 4:** Top five significant hits, if any, of selected Cmap queries within microarray platforms

Signatures	1st match	2nd match	3rd match	4th match	5th match
	Within ZF 21K (p-value cutoff 1/45 = 0.022)				
EE2_30ngL_Ovary_96hr	Self	FAD_Ovary	TRB_F_Brain	**EE2_Testis**	FAD_M_Brain
EE2_30ngL_M_Brain_48hr	Self	**EE2_Testis**	FAD_M_Brain	TRB_F_Brain	**EE2_Ovary**
EE2_30ngL_M_Liver_48hr	Self	TRB_F_Liver	**EE2_Testis**	O2_Testis	KET_M_Liver
EE2_30ngL_Testis_48hr	Self	**EE2_Ovary**	**EE2_M_Brain**	FIP_F_Brain	TRI_TestisLow
FAD_25ugL_F_Brain_48hr	Self	TRB_F_Brain	**FAD_M_Brain**	**FAD_Ovary**	EE2_Testis
FAD_25ugL_Ovary_96hr	Self	EE2_Ovary	**FAD_M_Brain**	TRI_TestisLow	TRB_F_Brain
FIP_5ugL_Testis_48hr	Self	**FIP_Ovary**	TRI_Testis	**FIP_M_Brain**	FLU_Testis
FLU_1700ugL_Ovary_48hr	Self	**FLU_Testis**	PRO_Testis	VIN_Ovary	KET_Ovary
MUS_500ugL_F_Brain_48hr	Self	VIN_Ovary	**MUS_M_Brain**	KET_M_Brain	VIN_Testis
MUS_500ugL_M_Brain_48hr	Self	**MUS_F_Brain**	VIN_Ovary	KET_M_Brain	KET_Ovary
O2_1mgL_Testis_4d	Self	**O2_Ovary**	EE2_M_Liver	**O2_Ovary**	VIN_Testis
PRO_500ugL_F_Brain_48hr	FLU_Testis	self	**PRO_Testis**	VIN_Ovary	KET_Ovary
PRO_500ugL_Ovary_48hr	Self	FLU_Ovary	FLU_Testis	KET_Ovary	**PRO_Testis**
TCDD_2nM_Embryo_6h	Self	**TCDD_Embryo**	TRI_Ovary	O2_Ovary	PRO_Testis
TRI_2500ugL_Testis_24hr	Self	VIN_Testis	VIN_Ovary	KET_Ovary	**TRI_TestisLow**
VIN_1000ugL_Ovary_48hr	Self	KET_Ovary	TRI_Testis	**VIN_Testis**	FIP_Ovary
VIN_1000ugL_Testis_48hr	Self	FLU_Testis	PRO_Testis	TRI_Testis	**VIN_Ovary**
	Within ZF 43K (p-value cutoff 1/39 = 0.026)				
BPA_0.01ugL_Ovary_96hr	Self	**BPA_Ovary**	**BPA_Ovary**	**BPA_Ovary**	**BPA_Embryo**
E2_1uM_Embryo_4dpf	self	**E2_M_Liver**	**GEN_Embryo**	**GEN_Embryo**	FLU_Embryo
EE2_0.65mgL_Embryo_48hr	**EE2_Embryo**	**EE2_Embryo**	**GEN_Embryo**	PARAB_Embryo	PYR_Embryo
E2_5ugL_M_Liver_48hrs	self	**E2_Embryo**	PARAB_Embryo	PARAB_Embryo	**GEN_Embryo**
GEN_2.4mgL_Embryo_48hr	self	**GEN_Embryo**	PARAB_Embryo	DBT_Embryo	diazepam_Brain
PKC412_40ugL_fish_6dpf	**PKC412_fish**	self	RDX_fry	diazepam_Brain	RDX_fry
PRO_500ugL_F_Brain_48hr	**PRO_Ovary**	linuron_Embryo	FLU_Ovary	**PRO_F_Brain**	linuron_Embryo
PRO_500ugL_Ovary_48hr	self	FLU_Ovary	linuron_Embryo	**PRO_F_Brain**	linuron_Embryo
RDX_7.5mgL_fry_96hr	self	**RDX_fry**	**RDX_fry**	**RDX_fry**	**RDX_fry**
BPA_8mgL_Embryo_48hr	self	**BPA_Embryo**	EE2_Embryo	BAA_Embryo	PRO_Embryo
diazepam_273ngL_brain_14d	self	**diazepam_Brain**	PKC412_fish	RDX_fry	RDX_fry
linuron_1.2mgL_Embryo_48hr	self	**linuron_Embryo**	PKC412_fish	BPA_Embryo	diazepam_Brain
PARAB_19.8mgL_Embryo_48hr	self	**PARAB_Embryo**	linuron_Embryo	linuron_Embryo	PYR_Embryo
PRO_1.7mgL_Embryo_48hr	self	**PRO_Embryo**	E2_Embryo	BPA_Embryo	PARAB_Embryo
	Within FHM 15K (p-value cutoff 1/132 = 0.0076)				
BIF_0.15ugL_Larvae_48hr	**BIF_Larvae**	self	TER_M_Brain	**Mixture_M_Brain**	KETTNTKET_Ovary
BIF_0.3ugL_Larvae_48hr	self	**CYP_Larvae**	**ESF_Larvae**	**ESF_Larvae**	**PER_Larvae**
CYP_1ugL_Larvae_48hr	self	VZ_Ovary	**PER_Larvae**	**CYP_larva**	RDX_Ovary
DES_1ngL_Liver_96h	**EE2_Liver**	self	PHE_High_Liver	**E2_X_M_Liver**	**DES_Liver**
E2_4ugL_M_Liver_14d	self	**E2_M_Liver**	**EE2_Liver**	**DES_Liver**	**ElyEFF_Liver**
EE2_25ngL_Liver_72h	**E2_M_Liver**	self	**E2_X_M_Liver**	**DES_Liver**	**ElyEFF_Liver**
GEM_600ugL_Ovary_8d	self	**GEM_Ovary**	TrbEE2TrbEE2_Ovary	RochDS_Ovary	TrbEE2TRB_Ovary
PHE_High_Liver_48hr	self	**PHE_Med_Liver**	ElyEFF_Liver	ElyUS_Liver	PFCs_Low_Liver
TRB_30ngL_Ovary_24h	self	FLU_Ovary	TrbTCCTCC_Ovary	GEM_Ovary	GEM_Ovary
TER_57.5ugL_M_Brain_72h	self	**Mixture_M_Brain**	PER_M_Brain	BIF_Larvae	KET_Ovary

Only signatures connected to multiple conditions are included. Where similar signatures exist for a condition, only one is listed. ROGL hits are ranked by their adjusted connectivity scores and filtered by a p-value cutoff of 1/(number of sets of ROGLs). Informative connections, defined as a signature and one of its top five ROGL hits sharing the same or similar class of chemicals, are highlighted in bold. Self: a query signature and the connected ROGLs originated from the same experiment

Beyond single chemical exposures, Cmap was also effective in discriminating chemical mixtures. For example, when fish were exposed to a mixture of two different chemicals (Mixture_M_Brain; containing terbufos and permethrin), this mixture had barely detectable effects on gene expression, so no signature could be constructed. Still, its ROGLs were informative, connecting to the signatures of both terbufos and bifenthrin from other independent experiments. Bifenthrin is another pyrethroid insecticide similar to permethrin. Notable was the fact that the bifenthrin signature here originated from whole fish larvae while the ROGLs of terbufos/permethrin mixture came from adult brain tissue. At the concentrations used in the exposure, permethrin had very little effect on gene expression, resulting in a relatively uninformative signature consisting of only six gene probes. As ROGLs, however, permethrin also formed strong connection with terbufos and bifenthrin.

The microarray data from previous studies of water conditions near several municipal WWTPs across the USA provided a “real world” assessment of Cmap performance (Table 5). Several observations were notable. There were considerable similarities among the water conditions near these WWTPs as reflected in their fish ROGLs. These similarities were observed both within locations (e.g., upstream, effluent, downstream) and across geographic locations. Second, there were high agreements between Cmap and chemical analyses from those studies with regard to the occurrence of known estrogenical chemicals, such as E2 and EE2 [29, 30]. For example, the water samples near WWTPs of San Diego (EFFHa, EFFLa) and Los Angeles (EFFHb), California were both reported to be estrogen-positive [29]. Their estrogenic identities were confirmed by Cmap when they were examined either as query signatures (Table 5) or ROGLs (not shown). Various types of estrogenic chemicals were also found near the WWTPs of Ely, Hutchinson, and Rochester, MN [30]. By Cmap, estrogen-associated ROGLs were also connected to the signatures of these water samples, with connectivity scores ranked at 10th (Ely effluent, Ely downstream), 16th (Ely upstream, Hutchinson effluent), 13th (Hutchinson downstream), 18th (Rochester downstream), 11th (Rochester effluent), and 12th (Rochester upstream). Measured against a p-value cutoff of 1/132 = 0.0076, all these estrogen connections were statistically highly significant. Finally, the sediment samples, and presumably the surrounding water, near the WWTPs in Ely and Hutchinson were positive for a number of PAHs (e.g. anthracene, phenanthrene, pyrene) and an organophosphate insecticide (chlorpyrifos) [31]. The query signatures from both locations ranked phenanthrene with high connectivity scores. With the same MIE (acetylcholinesterase inhibitor) shared between terbufos and chlorpyrifos, a strong connectivity was also found between a terbufos signature and the ROGLs of Ely downstream (6th), Ely effluent (8th), Hutchinson downstream (14th), and Hutchinson effluent (16th).

**Table 5:** Cmap performance under “real world” conditions

Signatures (NCBI GEO accession)	1st – 2nd match	3rd-4th match	5th-6th match	7th-8th match	9th-10th match
EFFHa_5Perc_MaleLiver_14d (GSE29350)	self	BIF_Larvae	BIF_Larvae	Wastewater_Liver	FLU_50_Ovary
	EFFLa_M_Liver	HutDS_Liver	EFFHb_M_Liver	HutUS_Liver	PFCs_Mix_Liver
EFFHb_5Perc_MaleLiver_14d (GSE29350)	self	Wastewater_Liver	FAD_Ovary	TRI_Ovary	RDX_Liver
	E2_M_Liver	HutEFF_Ovary	VIN_Ovary	KET_Ovary	RDX_fry
ElyDS_999_Liver_4d (GSE49098)	self	HutEFF_Liver	RochUS_Liver	RochDS_Liver	PHE_Liver
	ElyEFF_Liver	ElyUS_Liver	HutUS_Liver	HutDS_Liver	E2_M_Liver
ElyEFF_999_Liver_4d (GSE49098)	self	ElyUS_Liver	PHE_Liver	RochDS_Liver	RochUS_Liver
	ElyDS_Liver	HutEFF_Liver	HutUS_Liver	PHE_Liver	E2_M_Liver
ElyUS_999_Liver_4d (GSE49098)	self	ElyDS_Liver	HutEFF_Liver	HutUS_Liver	BIF_Larvae
	ElyEFF_Liver	PFCs_Liver	EFFHa_M_Liver	RochDS_Liver	PFCs_Mix_Liver
HutchinsonDS_999_Liver_4d (GSE49098)	self	RochDS_Liver	HutEFF_Liver	ElyEFF_Liver	PFCs_Mix_Liver
	RochUS_Liver	ElyDS_Liver	HutUS_Liver	KET_Ovary	KET_Ovary
HutchinsonEFF_999_Liver_4d (GSE49098)	self	ElyDS_Liver	HutUS_Liver	HutDS_Liver	PHE_Liver
	ElyEFF_Liver	ElyUS_Liver	RochUS_Liver	RochDS_Liver	KET_Ovary
RochesterDS_999_Liver_4d (GSE49098)	self	HutUS_Liver	HutDS_Liver	ElyEFF_Liver	RochEFF_Liver
	RochUS_Liver	ElyDS_Liver	ElyUS_Liver	HutEFF_Liver	BIF_Larvae
RochesterEFF_999_Liver_4d (GSE49098)	HutUS_Liver	self	HutEFF_Liver	BIF_Larvae	PFCs_Mix_Liver
	ElyUS_Liver	RochDS_Liver	ElyDS_Liver	RochUS_Liver	PFCs_Liver
RochesterUS_999_Liver_4d (GSE49098)	self	RochDS_Liver	HutEFF_Liver	HutUS_Liver	ElyUS_Liver
	ElyDS_Liver	ElyEFF_Liver	HutDS_Liver	RochEFF_Liver	PFCs_Mix_Liver
Wastewater_999_Liver_48h (GSE37550)	self	CYP_Larvae	NA	NA	NA
	E2_M_Liver	surfaceH2O_Testis	NA	NA	NA

Fathead minnow samples were exposed to various water conditions near several waste water treatment plants in the USA either by field deployment or in a laboratory setting. Only those exposures generating a significant number of DEGs thus having query signatures available are listed. The top ten matched ROGLs are listed to provide a broad list of candidate chemicals. Waste water treatment plant locations: Ely, Hutchinson (Hut), Rochester (Roch), Minnesota; San Diego (Ha), Los Angeles (Hb), California; Gainesville (GSE37550), Florida. Abbreviations: EFF, effluent; US, upstream; DS, downstream. P-value cutoff 1/132 = 0.0076. Self: a query signature and the connected ROGLs originated from the same experiment

Cmap had a more limited success, ranging from 50 % to 85 %, across platforms and species based on a small number of conditions common to them (Table 2). In spite of varying performance between ZF 21K and ZF 43K in either direction, Cmap was able to connect query signatures to target ROGLs for all the conditions common to these two platforms: EE2/E2/genistein, flutamide, and prochloraz (Table 6, Additional file 4: Table S3). There was little difference in cross-platform performance between maximum signature size set at 100 and 500. Similar variation in the performance of Cmap was also observed across species. Among the conditions shared between FHM 15K and ZF 21K, and between FHM 15K and ZF 43K, were EE2/E2/DES/genistein, trenbolone, and RDX. With the exception of RDX and trenbolone, successful connections were made between query signatures and ROGLs for all other conditions.

**Table 6:** Top five significant hits, if any, of selected Cmap queries across microarray platforms and species

Signatures	1st match	2nd match	3rd match	4th match	5th match
	From ZF 21K to ZF 43K (p-value = 0.026; signature size average = 181, min = 15, max = 375)				
EE2_30ngL_M_Brain_48hr.sig.500.IDswap	LIN_Embryo	PRO_F_Brain	LIN_Embryo	**E2_M_Liver**	FLU_Ovary
EE2_30ngL_M_Liver_48hr.sig.500.IDswap	**E2_M_Liver**	RDX_fry	RDX_fry	RDX_fry	PKC412_fish
EE2_30ngL_M_Testis_48hr.sig.500.IDswap	**GEN_Embryo**	BPA_Ovary	BPA_Embryo	BPA_Ovary	DBT_Embryo
FLU_Ovary_48hr.sig.500.IDswap	**FLU_Embryo**	PKC412_fish	BPA_Ovary	PPL_Embryo	RDX_fry
FLU_Testis_48hr.sig.500.IDswap	LIN_Embryo	LIN_Embryo	**FLU_F_Ovary**	PKC412_fish	**FLU_Embryo**
PRO_F_Brain_48hr.sig.500.IDswap	E2_M_Liver	**PRO_F_Brain**	GEN_Embryo	FLU_Embryo	**PRO_Ovary**
PRO_Ovary_48hr.sig.500.IDswap	**PRO_Ovary**	FLU_Ovary	**PRO_F_Brain**		
PRO_Testis_48hr.sig.500.IDswap	E2_M_Liver	PRO_Ovary*	PRO_F_Brain*		
	From ZF 43K to ZF 21K (p-value = 0.022; signature size average = 134, min = 5, max = 310)				
E2_1uM_Embryo_4dpf.sig.500.IDswap	**EE2_Testis**	**EE2_M_Liver**	TRB_F_Liver	O2_Testis	O2_Ovary
E2_5ugL_M_Liver_4hrs.sig.500.IDswap	**EE2_M_Liver**	TRB_F_Liver	O2_Testis	O2_Testis	TCDD_Embryo
PRO_2mgL_Embryo_48hr.sig.500.IDswap	DMB_Embryo	tBHQ_Embryo	TRB_F_Brain	**PRO_F_Brain**	EE2_M_Liver
	From ZF 21K to FHM 15K (p-value = 0.0076; signature size average = 124, min = 9, max = 257)				
EE2_30ngL_Ovary_96hr.sig.500.IDswap	FAD_Ovary	**EE2_M_Brain**	EE2_M_Brain*		
EE2_30ngL_M_Liver_48hr.sig.500.IDswap	**EE2_Liver**	RochDS_liver	PHE_Liver	RochUS_liver	Stream_Liver
EE2_30ngL_Testis_48hr.sig.500.IDswap	PFCs_Liver	KTC_Ovary	PFCs_High_Liver	**RochEFF_liver**	
	From FHM 15K to ZF 21K (p- value = 0.022; signature size average = 110, min = 5, max = 272)				
DES_100ngL_Liver_96h.sig.500.IDswap	**EE2_M_Liver**	DNP_Embryo	DMB_Embryo	TCDD_Embryo	APM_Embryo
E2a_4ugL_M_Liver_14d.sig.500.IDswap	**EE2_M_Liver**	KET_F_Liver	DNP_Embryo	KET_M_Liver	TRI_Ovary
EE2_25ngL_Liver_72h.sig.500.IDswap	**EE2_M_Liver**	KET_M_Liver	KET_F_Liver	TRB_F_Liver	FLU_M_Testis
	From ZF 43K to FHM 15K (p-value = 0.0076; signature size average = 120, min = 6, max = 274)				
E2_M_Liver_4hrs.sig.500.IDswap	**ElyEFF_liver**	ElyDS_liver	PHE_Liver	**E2_M_Liver**	**DES_Liver**
GEN_Embryo_48hr.sig.500.IDswap	PFCs_Blood	KTC_Ovary	BIF_Larvae	**E2_M_Liver**	PER_Larvae
	From FHM 15K to ZF 43K (p-value = 0.026; signature size average = 139, min = 5, max = 363)				
DES_1ngL_Liver_96h.sig.500.IDswap	**E2_M_Liver**				
E2_4ugL_M_Liver_14d.sig.500.IDswap	**E2_M_Liver**	PKC412_fish	BPA_Embryo	**GEN_Embryo**	BPA_Embryo
EE2_25ngL_Liver_72h.sig.500.IDswap	**E2_M_Liver**	PKC412_fish	LIN_Embryo	BPA_Embryo	BDE_Embryo

Where similar signatures exist for a condition, only one of them is listed. ROGL hits are ranked by their adjusted connectivity scores and filtered by a p-value cutoff of 1/(number of sets of ROGLs). Informative connections, defined as a signature and one of its top five ROGL hits sharing the same or similar class of chemicals, are highlighted in bold. “*”, *p-*values slightly greater than the cutoffs

### Discovery of novel chemical connections

While the chemical identities of individual query signatures and their connected high-scored ROGLs enable an evaluation of Cmap performance, those connections that ranked high across query signatures also could provide novel insights into possibly shared MOAs and toxicity pathways among seemingly different chemicals. By individual platforms of ZF 21K, ZF 43K, and FHM 15K, the highest-scored connection was selected from all the significant ones for each distinct pair of chemicals, regardless of direction (i.e., two conditions linked to each other as either a signature or ROGLs), tissue type, dose, exposure duration or data origin, and visualized collectively in Cytoscape as a chemical network. With the recognition of likely confounding contributions from such factors as tissue type, this network provided a composite or “average” view of how chemicals relate to one another. Each chemical in this network became a node, with the connection between two nodes forming an edge. An edge was weighted by the connectivity score between the two connected chemicals. Such a network essentially provides a glimpse of chemical “neighborhood” as arranged by the similarities in the transcriptomic profiles among its chemical members.

**Fig. 1:**
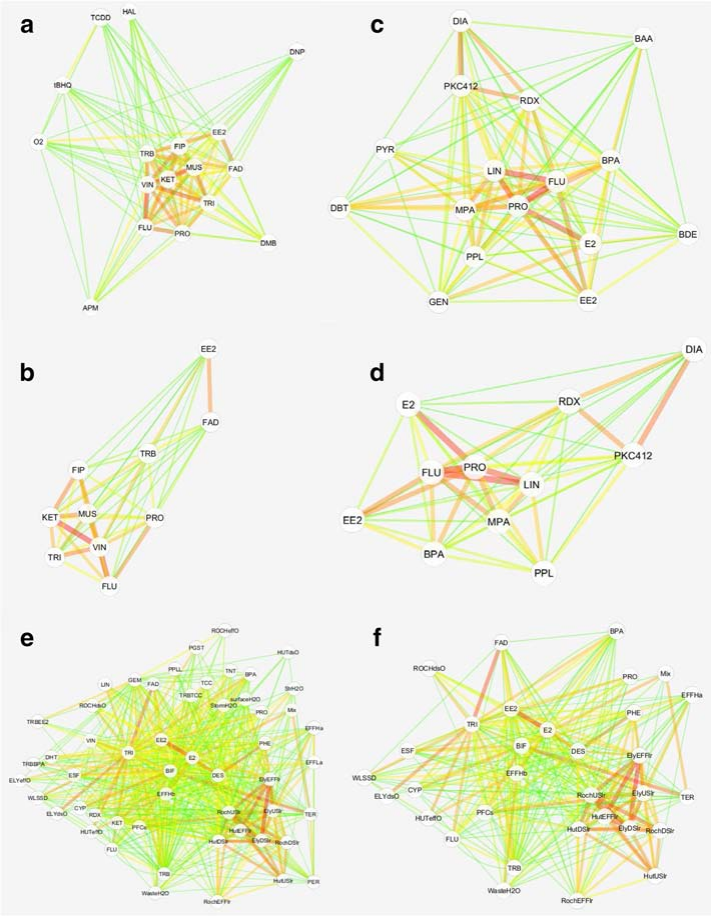
A network view of chemical-chemical connectivity based on fish samples profiled on various microarray platforms. Each treatment condition is represented as a node. Two nodes are connected by an edge weighted by their connectivity score. A shorter, darker, and wider edge between two nodes denotes a higher connectivity score. All connections shown are statistically significant. **a** ZF 21K: 117 connections among 17 treatment conditions; **b** ZF 21K: 45 connections among 10 nodes with each node containing a connectivity score of ≥ 10 in at least one connection; **c** ZF 43K: 110 connections among 16 treatment conditions; **d** ZF 43K: 54 connections among 11 nodes with each node containing a connectivity score of ≥ 10 in at least one connection; E) FHM 15K: 541 connections among 53 treatment conditions; F) FHM 15K: 293 connections among 32 nodes with each node containing a connectivity score of ≥ 10 in at least one connection

In ZF 21K where a total of 17 chemicals were linked, 10 confirmed HPG-active toxicants formed a tight cluster in the network while other chemicals that likely were not HPG-active spread out as distant outliers (Fig. 1a). A further differentiation of the 10 HPG-active toxicants could be made when they were selected to form their own sub-network based on achieving a minimum connectivity score (Fig. 1b). Under this scenario, there appeared to be two distinct groups: one made up of vinclozolin, ketoconazole, flutamide, trilostane, fipronil, muscimol, and prochloraz; and the other of EE2 and fadrozole. Trenbolone seemed to be unique as it shared substantial similarity to both groups. These observed clustering patterns were still clearly visible when both chemical and tissue type were considered together (Additional file 5: Figure S2). Beyond the contributions from chemical and sometimes tissue type, the structure of this network did not appear to coincide with other experimental conditions such as the lab origins of data.

In ZF 43K, there was also a distinct pattern in chemical connectivity (Fig. 1c, d). Prochloraz, flutamide, linuron, E2, and to the lesser extent, EE2, methylparaben, bisphenol A, formed a group of similar chemicals while RDX, PKC412, and diazepam formed another. Interestingly, the three confirmed estrogens; genistein, EE2, and E2; had relatively weak connections among themselves.

In FHM 15K, a number of interesting observations could also be made. Consistent with the earlier analysis by individual query signatures (Table 5), the water conditions near the three Minnesota WWTPs, as reflected in the transcriptomic profiles of deployed male fish, were indeed highly similar to one another not only across sites within a location, but also across locations (Fig. 1e, f; liver tissue of male fish exposed to water from Ely effluent, downstream, upstream: ElyEFFlr, ElyDSlr, ElyUSlr; Hutchinson effluent, downstream, upstream: HutEFFlr, HutDSlr, HutUSlr; Rochester effluent, downstream, upstream: RochEFFlr, RochDSlr, RochUSlr). These sites, all of which had detectable estrogens present [30], showed relatively strong connectivity with diethylstilbestrol (DES), E2, and EE2. However, in female fish samples exposed to these same sites (ELYeffO, ELYdsO, HUTdsO, HUTeffO, ROCHeffO, ROCHdsO), no detectable impact was found on gene expression, and perhaps not surprisingly, no obvious pattern in the connectivity among these conditions was observed. In a similar but independent study of WWTPs in San Diego (EFFHa, EFFLa) and Los Angeles (EFFHb), California [29], potential estrogenic properties as determined by chemistry were revealed in the current study by their significant connectivity to DES, E2, and EE2. Also notable is the fact that, probably due to the San Diego WWTP being a primary treatment plant and the Los Angeles WWTP a secondary treatment plant, their ROGLs were quite distinct. Finally, several pyrethroid insecticides (bifenthrin, BIF; cypermethrin, CYP; esfenvalerate, ESF), though seemingly far apart, also shared strong connectivity among themselves as indicated by their edge size and color. In the case of a mixture (Mix) of terbufos (TER) and permethrin (PER), the mixture was strongly connected to both terbufos and bifenthrin, but not to permethrin directly. Interestingly, permethrin was significantly linked to both terbufos and bifenthrin (Fig. 1e, Table 4).

**Fig. 2:**
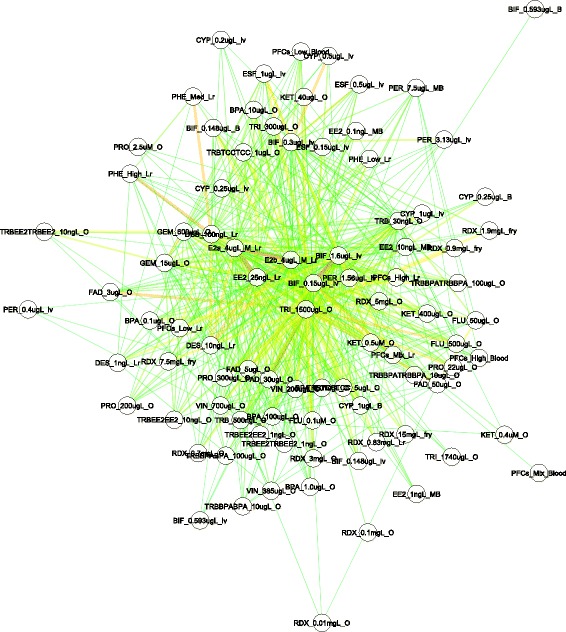
A network view of chemical-chemical connectivity based on fish samples profiled on FHM 15K. Only treatments with multiple doses are included. Each treatment condition containing the information of both dosage and tissue type is represented as a node. Two nodes are connected by an edge weighted by their connectivity score. A shorter, darker, and wider edge between two nodes denote a higher connectivity score. All connections shown are statistically significant. Tissue types and gender: T, testis; O, ovary; B, brain; Lr, liver; lv, larvae; M, male

Connectivity among chemicals appeared to be modulated by the intensity of their elicited transcriptomic responses, which are dependent on chemical dose, treatment duration, fish tissue type, and life stage. When only fathead minnow treatments with multiple doses of the same tissue type were considered, the nodes of the same chemical but with various doses were scattered throughout the network, with different numbers of neighbors (directly connected nodes) at different connectivity strength (Fig. 2). For a given node, the number of its neighbors measures its connectivity in a network. For instance, the two nodes representing two independent but concurrent bifenthrin exposure experiments with larvae (BIF_0.148ugL, BIF_0.593ugL) had 7 and 4 neighbors respectively, as determined by Cytoscape. Their closest neighbors by connectivity score, bifenthrin (BIF_0.15ugL, 7.2) and trilostane (TRI_1500ugL, 4.8) respectively, were also different. In other words, depending on dosage, the network neighborhood of a chemical could be altered to some extent. Similar observations were also made in other conditions such as bisphenol A with ovary (BPA_0.01ugL, BPA_0.1ugL, BPA_1.0ugL, BPA_10ugL, and BPA_100ugL; 9, 4, 7, 9, 11 neighbors), DES with liver (DES_1ngL, DES_10ngL, DES_100ngL; 10, 8, and 47 neighbors), and fadrozole with ovary (FAD_3ugL, FAD_5ugL, FAD_30ugL, FAD_50ugL; 8, 10, 8, 11 neighbors).

## Discussion

Fish Cmap provides a data-driven approach for applying transcriptome profiling technology to the assessment of exposure, relative toxicity, and grouping of chemicals. The findings in this study have demonstrated the effectiveness of this approach to make connections among chemical conditions associated with a query signature and a set of ROGLs from independent experiments, especially when both are from the same microarray platform/species. Like any other query-database applications, its power is a function of coverage: the more chemical/biological conditions ROGLs in a database are linked to, the more likely a query signature will make an informative connection. Along with the rapid increase of transcriptomics data in public repositories and the expansion of this fish reference database in the future, fish Cmap should find increasing applications in ecotoxicology.

The performance of fish Cmap ultimately depends on MOAs/toxicity pathways shared among chemicals. For a given query signature and its target ROGLs, the connectivity strength is determined by both the direction in change (up, + or down, −) and ranking in magnitude of the selected DEGs therein (typically 10 to 100). In the current study, the direction of DEGs was considered in both signatures and ROGLs, but the ranking was considered only in ROGLs. In this configuration, a connectivity score is maximized when the signs of DEGs in a query signature perfectly match their counterparts in the target ROGLs, and these DEGs are ranked high in the latter [24]. Arguably, the direction of change of a DEG and its relative rank are not as sensitive as magnitude to extraneous factors such as exposure intensity (chemical dosage and duration) and “random” noise (natural variation; Wang et al. [16]), thus are more reflective of the underlying chemical MOA. In theory, a treatment condition must be of sufficient intensity to have a significant impact on fish transcriptome and enable subsequent Cmap. The relationship between connectivity strength and the magnitude of treatment effect as indicated by DEGs was examined in the current study by several measures of individual chemical conditions: relative transcriptome impact (RTI, the percentage of a transcriptome determined as DEGs), the average LogFC in absolute values over all DEGs, and total number of DEGs. There were no clear relationships for connectivity score (normalized to signature size), RTI, and average LogFC (Additional file 6: Figure S3A, S3B, S3C) among 106 chemical conditions with a RTI ≥ 0.001. However, a further examination within FHM 15K, which has 13 signatures each containing less than 10 probes, revealed that connectivity scores (un-normalized) tended to increase as a function of total number of DEGs (Additional file 7: Table S4). All 11 unsuccessful queries (measured by p-value of 0.0076 in top five hits) occurred in conditions in which each had less than 100 DEGs in total. On the other hand, many chemical conditions with very few detectable DEGs were actually still able to yield informative ROGLs to allow appropriate connections with relevant query signatures. These pieces of evidence thus reinforce the importance of DEGs’ directions of change and rankings to chemical connectivity in comparison to their LogFC, and the importance of guarding against false positives when a treatment condition has only minimal effects. Furthermore, Cmap connectivity should, in theory, be largely driven by chemical MOAs because both signatures and ROGLs were generated within the same tissue type between a treatment condition and corresponding experimental controls. Indeed, Cmap performed very well in connecting query signatures to their target ROGLs, especially within platforms/species. These connections were often made across experimental origins, chemical classes, complexity of exposures, and tissue types. As illustrated by fish samples from ZF 21K, when the connections of associated chemicals and tissue types were visualized simultaneously in a network, many nodes were distributed based on chemical conditions, not tissue types (Additional file 5: Figure S2A, S2B). However, it is evident that, in some cases, tissue type did contribute significantly to chemical connectivity. Such a confounding effect is probably a function of chemical, dosage, and tissue type.

Besides making a greater amount of public fish transcriptomic data available for Cmap, the primary significance in attempting interspecific Cmap lies in the prospect of extrapolating chemical toxicity across fish species. This is critical because it is impossible to test the toxicity of all chemicals of possible concern in all fish species. There are two potential limiting factors affecting the success of inter-specific (inter-platform) Cmap. One is genome annotation and probe mapping to identify orthologous/equivalent probes. The other is the conservation of toxicity pathways/MOAs between species. Given a very small number of conditions common across platforms and species in this study, it is difficult to assess Cmap performance across platforms/species reliably. However, generally low percentages of cross-mapped probes (62 % of ZF 21K vs 37 % of ZF 43K; 43 % of ZF 21K vs 45 % of FHM 15K; 38 % of ZF 43K vs 65 % of FHM 15K) suggest that a substantial loss of information was probably responsible in part for the relatively poor performance in the preliminary Cmap across platforms/species. With the relatively recent divergence between zebrafish and fathead minnow [15], broad conservation of molecular pathways among animal species [32–38], and high degree of genome conservation even between zebrafish and human [39], it seems reasonable to hypothesize that toxicity pathways are well conserved between these two small fish species. Indeed, a recent study of their transcriptomes provided strong evidence in this regard [16]. If this is the case, conservation of toxicity pathways should not be the primary issue in the performance of interspecific Cmap in the current study.

In addition to being able to connect the same chemicals underlying a query signature and its target ROGLs, Cmap also provided novel insights into some seemingly different chemicals that may possess similar MOAs. For those studies based on ZF 21K, Cmap grouped several well-characterized HPG-active toxicants together (Fig. 1a) [18], and further differentiated them into two sub-groups (Fig. 1b). One subgroup consisted of EE2 and fadrozole only; the other included vinclozolin, ketoconazole, flutamide, trilostane, fipronil, muscimol, and prochloraz. Trenbolone appeared to be an intermediate between the two subgroups. These clustering patterns did not correlate with extraneous factors such as tissue type or lab origins of data. For example, four different research groups produced data behind these nodes: oxygen, haloperidol and 10 other HPG-active toxicants; tetrachlorodibenzo-p-dioxin (TCDD); TCDD, tert-butylhydroquinone; dinitrophenol, dimethoxybenzene, and azinphosmethyl. Rather, the distribution patterns of these chemicals are more in line with their MOAs, some of which involve multiple MIEs. As an aromatase inhibitor, fadrozole blocks the transformation of testosterone to E2, the primary endogenous ligand for the estrogen receptor (ER), so it is reasonable that EE2 and fadrozole would cluster together. Further, since testosterone is a ligand for the androgen receptor (AR), it is quite conceivable to envision the same genes being activated by EE2, fadrozole, and trenbolone, a synthetic AR agonist [40, 41]. A similar argument could also be invoked to explain the shared MOAs of vinclozolin, flutamide, ketozonazole, and to a lesser extent, prochloraz, because of their common impact again on AR and aromatase: vinclozolin, flutamide, prochloraz are all AR antagonists, while ketoconazole and prochloraz are both aromatase inhibitors [18]. Note that fadrozole also shared strong connectivity with ketoconazole and prochloraz. There are other examples, as well, of the Cmap analysis highlighting chemicals that impact the same molecular target. For example, although fipronil and muscimol have opposite effects on gamma-aminobutyric acid (GABA) receptor, one being an antagonist while the other an agonist, they are identified as substantially similar. Between muscimol (MUS_500ugL_femaleBrain) as a signature and fipronil (FIP_5ugL_femaleOvary) as ROGLs, they reached a high connectivity score of 16.8 (ranged 2.5-23.6 in ZF 21K), with the corresponding average LogFC of only 0.42 and 0.33. In other words, these DEGs changed largely in the same direction and ranked high in both muscimol and fipronil-treated fish, despite the differences in tissue being examined.

Novel insights on chemical MOAs were gained as well from studies based on ZF 43K and FHM 15K. For ZF 43K-based studies, chemicals acting as AR antagonists, prochloraz and flutamide, had MOAs similar to those of ER agonists, E2 and EE2 (Fig. 1c, d), suggesting that, at some level, anti-androgens and ER agonists are somewhat functionally equivalent biologically. Indeed, bisphenol A, a compound known to exhibit both estrogenic and anti-androgenic effects [42, 43], showed substantial connectivity with prochloraz (score 12.0), flutamide (10.8), EE2 (8.9), and E2 (6.8). The strength of these connections represents 37-65 % of the maximum connectivity score observed in the ZF 43K-based studies. Linuron, a phenylurea herbicide and a confirmed anti-androgen [44], also formed strong connections to prochloraz and flutamide. Methylparaben, a common preservative in cosmetic products, has been shown to possess both estrogenic and anti-androgenic activities [45].

For FHM 15K-based studies (Fig. 1e, f), Cmap demonstrated its effectiveness in discriminating chemical exposures across a range of complexities. For single chemical exposures, strong connectivity was found among several MOA-based classes of chemicals including ER agonists (EE2, E2, DES), neuro-toxins (bifenthrin, permethrin, cypermethrin, esfenvalerate, terbufos), and inhibitors of steroidgenic enzymes 3β-HSD and aromatase (trilostane, fadrozole). Phenanthrene, a PAH, was also linked to DES, suggesting its possible estrogenicity.

Also notable is the fact that when a chemical condition has little detectable transcriptomic effect perhaps due to issues such as effective dosage, sample size, and statistical stringency, its ROGLs still could be informative and capable of connecting with appropriate query signatures. This was the case in a mixture of permethrin and terbufos, where, in the absence of its own signature and a sizeable signature from permethrin alone, its ROGLs could still connect with bifenthrin and terbufos. And so did the ROGLs from permethrin. Other than both being neuro-toxins but with different MIEs, bifenthrin and terbufos are not known to share any other mechanisms underlying their connectivity. Perhaps a more striking revelation came from male fathead minnow liver samples exposed to the effluents near several WWTPs in Minnesota, which contained a complex array of chemicals including several known estrogens, PAHs, and a neuro-toxin [30, 31]. In spite of being located in very different ecological environment (non-agricultural, agricultural, urban), the effluents of these plants were remarkably similar to one another as measured by their common impact on fish transcriptomes. Quite possibly, the main drivers behind such a similarity include PAHs, natural and synthetic ER agonists, as well as other pollutants, rather than agricultural chemicals. There was, in fact, substantial connectivity between the effluent samples and several single chemical studies with known PAHs (maximum score 10.5) and estrogens (maximum score of 9.4) as compared to the observed score range of 2.6 to 19.9 for FHM 15K (Fig. 1e, f). These same effluents, however, had hardly any effect on the ovaries of female fish samples treated in the same study; some of their representative nodes present in the network were widely scattered. Thus, across the research based on the three platforms, ZF 21K, ZF 43K, and FHM 15K, Cmap has demonstrated its effectiveness in not only connecting the same chemical conditions underlying query signatures and ROGLs, but also establishing novel connectivity among seemingly different chemicals based on shared MOAs.

Connectivity among chemicals is a function of their shared transcriptomic profiles, which in turn are likely modulated by the dose and duration of a treatment on the targeted fish tissue at a given life stage. Conceivably, a varying number of genes and pathways could become perturbed by the same chemical under different conditions, leading to a different degree of overlap among MOAs. This hypothesis is supported by the findings in the current study, where the same chemical tested at multiple doses had different number of neighbors in a network of chemicals. Similar observations of dose-dependent, differential transcriptomic responses were also reported recently for chemicals in human cell cultures and fish [46, 47]. Such a dependency between chemical dose and transcriptomic response have both scientific and practical implications for applying Cmap in ecotoxicology. Scientifically, a chemical MOA could then be considered as consisting of both core and peripheral toxicity pathways, which may be defined by their responsiveness as a function of exposure intensity (dosage and duration), the specificity to a given chemical or tissue type, or the importance to the integrity of their larger biological network. The earliest responders at the lowest exposure intensity may not be necessarily those most critical to an organism’s biological integrity. A better delineation of toxicity pathways in this regard should help to inform the assessment of relative toxicity among chemicals and their risks to ecosystems. For more practical application of Cmap in exposure assessment, however, those chemical-specific, earliest responsive pathways at the lowest exposure intensity are likely to be diagnostically useful in biomonitoring.

## Conclusions

In summary, fish Cmap built on a very large collection of public and private GEPs from zebrafish and fathead minnow performed well in this study, particularly when conducted within the same platforms/species. When a query signature from samples of interest was made against a reference database of ROGLs, informative connections were established at high success rates when both shared the same chemical conditions. In other words, Cmap provides an easily scalable framework for a simple query signature selected from DEGs to function as an exposure biomarker, without going through a typical time-consuming process of development and validation. More importantly, as demonstrated in this study, a large reference database of ROGLs also enables a query signature to cross interrogate other chemical conditions with overlapping MOAs, leading to novel groupings and subgroupings of seemingly unrelated chemicals at a finer resolution. By this approach, for example, the estrogenic and PAH identities of largely uncharacterized water samples near several WWTPs were confirmed, suggesting its future potential in real world applications. For toxicity extrapolation across fish species, however, a sufficient number of GEPs linked to chemical conditions common to multiple fish species are needed in the future in order to conduct a more thorough feasibility study of interspecific Cmap.

## Availability of supporting data

The data sets supporting the results of this article are available in the NCBI GEO repository, with the accession numbers GSE38070, GSE60202, GSE70807, and GSE70936. Additional datasets downloaded from GEO are listed in Additional file 1: Table S1.

## Additional files

Additional file 1: Table S1. A complete list of microarray data used in the current study. (XLSX 111 kb) Additional file 2: Table S2. A complete list of query signatures constructed in the current study. Signatures were named after fish sample treatment conditions by the order of chemical, dose, gender, tissue, and exposure duration. Some conditions may each have multiple signatures generated because of either repeated experiments or different parameters in analysis. (XLSX 19 kb) Additional file 3: Figure S1. Relative transcriptomic impact of various treatment conditions in fathead minnow. Effects are measured by the percentages of transcriptome determined to be DEGs. (PPTX 39 kb) Additional file 4: Table S3. Cmap across ZF 21K and ZF 43K with the maximum signature size of 100 probes. (DOCX 14 kb) Additional file 5: Figure S2. A network view of chemicals and tissue types based on samples profiled on ZF 21K. Each chemical treatment condition and the tissue profiled is represented as a node. Two nodes are connected by an edge weighted by their connectivity score. A shorter, darker, and wider edge between two nodes denote a higher connectivity score. All connections shown are statistically significant. A) 40 nodes with 573 connections; B) 30 nodes with 380 connections with each node having a minimum connectivity score of 10 in at least one of its connections. Tissue types: T, testis; O, ovary; B, brain; Lr, liver. (PDF 37 kb) Additional file 6: Figure S3. Relationships between connectivity strength and DEGs. A total of 106 chemical conditions with relative transcriptomic impact ≥ 0.001 were considered. Connectivity scores were normalized to the size of respective query signatures. A) average LogFC vs connectivity scores; B) average logFC vs relative transcriptomic impact (RTI, percentage of transcriptome as DEGs); C) connectivity scores vs RTIs. (PPTX 49 kb) Additional file 7: Table S4. Relationship between total number of DEGs and connectivity score among treatment conditions within FHM 15K. (XLSX 24 kb)

## Abbreviations

Agilent 013223: ZF 21K
Agilent 015064: ZF 21K
Agilent 019161: ZF 43K
Agilent 019597_036574: FHM 15K
APM: azinphos-methyl
BAA: benz(a)anthracene
BDE: decabromodiphenyl ether
BIF: bifenthrin
BPA: bisphenol A
CYP: cypermethrin
DBT: dibenzothiophene
DES: Diethylstilbestrol
DHT: Dihydrotestosterone
DIA: Diazepam
DMB: 1,4-dimethoxybenzene
DNP: 2,4-dinitrophenol
E2: 17ß-estradiol
EE2: ethniyl estradiol
EFFHa: Effluent of WWTP in San Diego, California
EFFHb: Effluent of WWTP in Los Angeles, California
EFFLa: Effluent of WWTP in San Diego, California
ELYds: Downstream of WWTP in Ely, Minnesota (ovary tissue sampled)
ElyDS: Downstream of WWTP in Ely, Minnesota (liver tissue sampled)
ELYeff: Effluent of WWTP in Ely, Minnesota (ovary tissue sampled)
ElyEFF: Effluent of WWTP in Ely, Minnesota (liver tissue sampled)
ElyUS: Upstream of WWTP in Ely, Minnesota (liver tissue sampled)
ESF: esfenvalerate
FAD: fadrozol
FIP: fipronil
FLU: flutamide
GEM: Gemfibrozil
GEN: genistein
HAL: Haloperidol
HUTds: Downstream of WWTP in Hutchinson, Minnesota (ovary tissue sampled)
HutDS: Downstream of WWTP in Hutchinson, Minnesota (liver tissue sampled)
HUTeff: Effluent of WWTP in Hutchinson, Minnesota (ovary tissue sampled)
HutEFF: Effluent of WWTP in Hutchinson, Minnesota (liver tissue sampled)
HutUS: Upstream of WWTP in Hutchinson, Minnesota (liver tissue sampled)
KET: ketochonazole
LIN: Linuron
LSW: Lake Superior water
MPA: methylparaben
MUS: muscimol
O2: oxygen
PER: permethrin
PFC: perflorinated chemicals
PGST: Progesterone
PHE: Phenanthrene
PKC412: protein kinase C inhibitor 412
PPL: Propanil
PPLL: Propranolol
PRO: prochloraz
PYR: Pyrene
RDX: cyclotrimethylenetrinitramine
ROCHds: Downstream of WWTP in Rochester, Minnesota (ovary tissue sampled)
RochDS: Downstream of WWTP in Rochester, Minnesota (liver tissue sampled)
ROCHeff: Effluent of WWTP in Rochester, Minnesota (ovary tissue sampled)
RochEFF: Effluent of WWTP in Rochester, Minnesota (liver tissue sampled)
RochUS: Upstream of WWTP in Rochester, Minnesota (liver tissue sampled)
stormH2O: storm water
strH2O: stream water
TBHQ: tert-Butylhydroquinone
TCC: Triclocarban
TCDD: 2,3,7,8-tetrachlorodibenzo-p-dioxin
TER: Terbufos
TNT: 2,4,6-trinitrotoluene
TRB: trenbolone
TRI: trilostane
VIN: vinclozolin
wasteH2O: waste water
WLSSD: Effluent, Western Lake Superior Sanitary District, Duluth, Minnesota
WWTP: waste water treatment plant
