# Supplementary material for: Fish connectivity mapping: linking chemical stressors by their mechanisms of action-driven transcriptomic profiles
Source: BMC Genomics. 2016 Jan 28;17:84. doi: 10.1186/s12864-016-2406-y (PMC4730593; doi:10.1186/s12864-016-2406-y)
Supplement: Additional file 3: Figure S1. — Relative transcriptomic impact of various treatment conditions in fathead minnow. Effects are measured by the percentages of transcriptome determined to be DEGs. (PPTX 39 kb) [file 12864_2016_2406_MOESM3_ESM.pptx]

## Slide 1
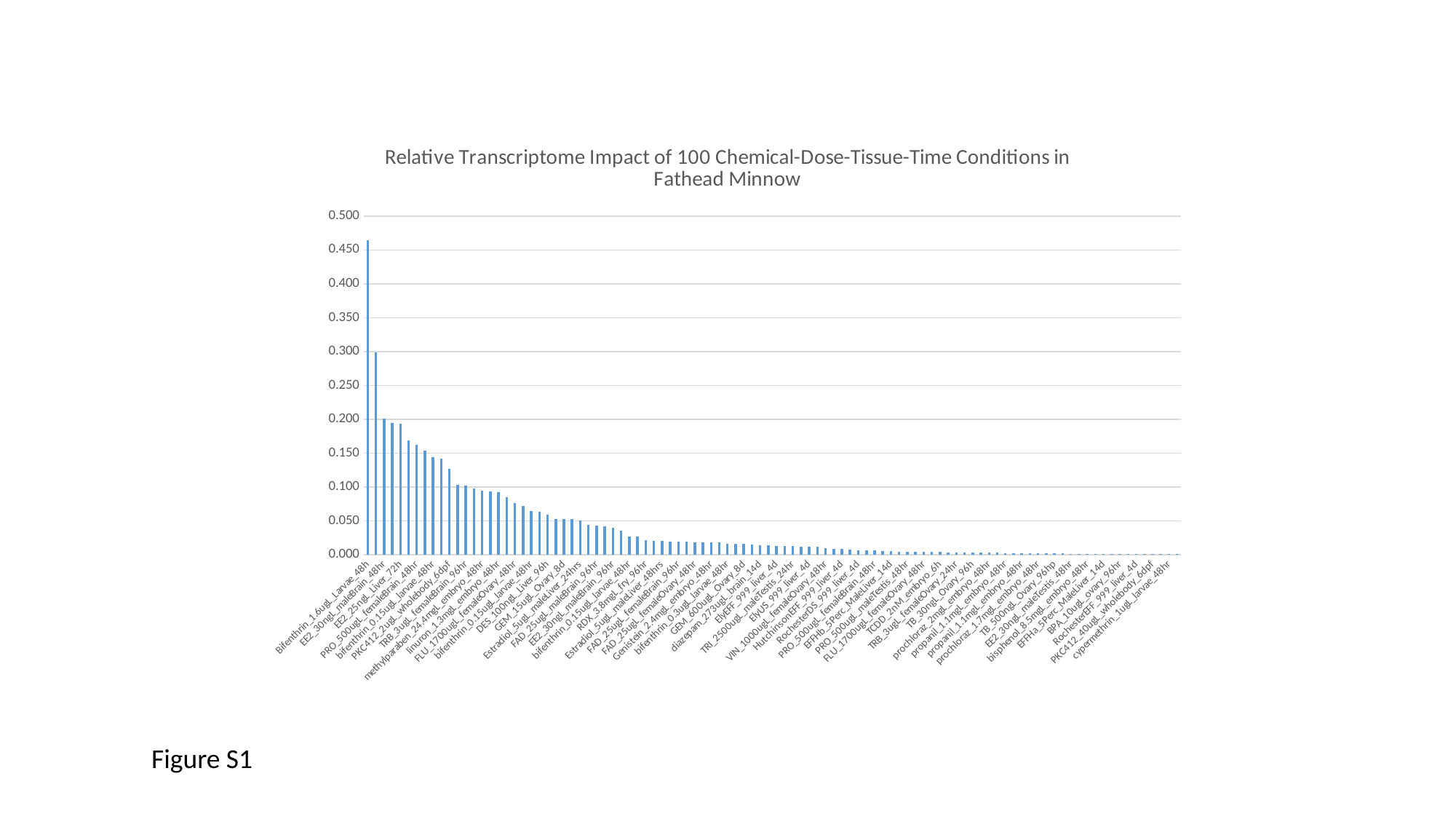

### Chart: Relative Transcriptome Impact of 100 Chemical-Dose-Tissue-Time Conditions in Fathead Minnow
| Category | RTI |
|---|---|
| Bifenthrin_1.6ugL_Larvae_48h | 0.4645429196172441 |
| EE2_30ngL_maleLiver_48hr | 0.2988602000465225 |
| EE2_30ngL_maleBrain_48hr | 0.20120958362409863 |
| bifenthrin_0.15ugL_larvae_48hr | 0.19450741238540392 |
| EE2_25ngL_Liver_72h | 0.19336395915665308 |
| FAD_25ugL_femaleOvary_48hr | 0.1682716910909514 |
| PRO_500ugL_femaleBrain_48hr | 0.1618050709467318 |
| FAD_25ugL_femaleBrain_48hr | 0.1536636427076064 |
| bifenthrin_0.15ugL_larvae_48hr | 0.14383437982707778 |
| EE2_30ngL_maleTestis_48hr | 0.14147476157245872 |
| PKC412_2ugL_wholebody_6dpf | 0.1267343990092425 |
| PKC412_2ugL_wholebody_6dpf | 0.1026534871453799 |
| TRB_3ugL_femaleBrain_96hr | 0.10230286113049547 |
| E2b_4ugL_MaleLiver_14d | 0.09771173066806944 |
| methylparaben_24.4mgL_embryo_48hr | 0.09444304290989153 |
| FLU_1700ugL_maleTestis_24hr | 0.09355664107932077 |
| linuron_1.3mgL_embryo_48hr | 0.09244776735545719 |
| EE2_30ngL_femaleOvary_96hr | 0.08508955571063039 |
| FLU_1700ugL_femaleOvary_48hr | 0.07573854384740637 |
| DES_100ngL_Liver_96h | 0.07226459758022094 |
| bifenthrin_0.15ugL_larvae_48hr | 0.06483580412846797 |
| FLU_1700ugL_maleTestis_48hr | 0.06317748313561293 |
| DES_100ngL_Liver_96h | 0.05898211467648606 |
| RDX_15mgL_fry_96hr | 0.053115611311148316 |
| GEM_15ugL_Ovary_8d | 0.052998421883219356 |
| linuron_1.3mgL_embryo_48hr | 0.05215237483659381 |
| Estradiol_5ugL_maleLiver_24hrs | 0.05004242827328395 |
| tBHQ_10uM_embryo_6h | 0.04419632472668062 |
| FAD_25ugL_maleBrain_96hr | 0.043405443126308445 |
| PRO_500ugL_femaleOvary_48hr | 0.04238194929053268 |
| EE2_30ngL_maleBrain_96hr | 0.03963712491277041 |
| Genistein_2.4mgL_embryo_48hr | 0.03575442056739215 |
| bifenthrin_0.15ugL_larvae_48hr | 0.027282392826335534 |
| FAD_25ugL_femaleOvary_96hr | 0.027029541753896255 |
| RDX_3.8mgL_fry_96hr | 0.021466412861500354 |
| VIN_1000ugL_maleTestis_24hr | 0.020702488950918817 |
| Estradiol_5ugL_maleLiver_48hrs | 0.020503176386945852 |
| FIP_5ugL_maleTestis_48hr | 0.019725517562223772 |
| FAD_25ugL_femaleBrain_96hr | 0.019167248197255175 |
| KET_370ugL_femaleOvary_24hr | 0.01898115840893231 |
| FAD_25ugL_femaleOvary_48hr | 0.018236799255640847 |
| HutchinsonDS_999_liver_4d | 0.018148342977380327 |
| Genistein_2.4mgL_embryo_48hr | 0.018026282595234273 |
| methylparaben_19.8mgL_embryo_48hr | 0.01772813797215788 |
| bifenthrin_0.3ugL_larvae_48hr | 0.016529920359485646 |
| VIN_1000ugL_maleTestis_48hr | 0.016422423819492906 |
| GEM_600ugL_Ovary_8d | 0.016175697001578115 |
| E2a_4ugL_MaleLiver_14d | 0.01499210941609679 |
| diazepam_273ugL_brain_14d | 0.014402678714767332 |
| RDX_7.5mgL_fry_96hr | 0.013921060477490081 |
| ElyEFF_999_liver_4d | 0.013282482903734877 |
| Terbufos_57.5ugL_MaleBrain_72h | 0.013216728037874803 |
| TRI_2500ugL_maleTestis_24hr | 0.01270062805303559 |
| Estradiol_5ugL_maleLiver_12hrs | 0.01210925853725661 |
| ElyUS_999_liver_4d | 0.011770120988953182 |
| EE2_30ngL_maleBrain_48hr | 0.011584089323098396 |
| VIN_1000ugL_femaleOvary_48hr | 0.009444056757385438 |
| MUS_500ugL_femaleBrain_48hr | 0.00837404047452896 |
| HutchinsonEFF_999_liver_4d | 0.008087848500789058 |
| RochesterUS_999_liver_4d | 0.007824829037348763 |
| RochesterDS_999_liver_4d | 0.006575486586007364 |
| ElyDS_999_liver_4d | 0.006575486586007364 |
| PRO_500ugL_femaleBrain_48hr | 0.0059170240579776625 |
| TRI_1500ugL_Ovary_48hp | 0.005391899000526039 |
| EFFHb_5Perc_MaleLiver_14d | 0.0053261441346659655 |
| PRO_500ugL_femaleOvary_48hr | 0.004770313969222301 |
| PRO_500ugL_maleTestis_48hr | 0.0043265875785066295 |
| MUS_500ugL_maleBrain_48hr | 0.004280065131425913 |
| FLU_1700ugL_femaleOvary_48hr | 0.004174024723069513 |
| TB_30ngL_Ovary_24h | 0.003879537085744345 |
| TCDD_2nM_embryo_6h | 0.003861363107699465 |
| KET_370ugL_femaleOvary_48hr | 0.0034891835310537334 |
| TRB_3ugL_femaleOvary_24hr | 0.0031635264014887184 |
| FIP_5ugL_femaleOvary_48hr | 0.0031635264014887184 |
| TB_30ngL_Ovary_96h | 0.002958968963703314 |
| linuron_1.2mgL_embryo_48hr | 0.002752104213012866 |
| prochloraz_2mgL_embryo_48hr | 0.002729170011237759 |
| bisphenol_8mgL_embryo_48hr | 0.002729170011237759 |
| propanil_1.1mgL_embryo_48hr | 0.0025227621952617937 |
| DES_1ngL_Liver_96h | 0.0019068911099421357 |
| propanil_1.1mgL_embryo_48hr | 0.0018347361420085774 |
| TB_30ngL_Ovary_192h | 0.0017753813782219884 |
| prochloraz_1.7mgL_embryo_48hr | 0.0017659335366832557 |
| FLU_50ugL_Ovary_96h | 0.0017096265123619147 |
| TB_500ngL_Ovary_96hp | 0.001643871646501841 |
| Estradiol_5ugL_maleLiver_4hrs | 0.0016283283260326124 |
| EE2_30ngL_maleTestis_48hr | 0.0015817632007443592 |
| TB_30ngL_Ovary_96hp | 0.0013808521830615465 |
| bisphenol_8.5mgL_embryo_48hr | 0.0012843152994060042 |
| diazepam_273ngL_brain_14d | 0.001146710088755361 |
| EFFHa_5Perc_MaleLiver_14d | 0.001117832719621252 |
| O2_1mgL_Testis_4d | 0.0009304489416143289 |
| BPA_10ugL_ovary_96hr | 0.0008026970621287526 |
| TB_500ngL_Ovary_48h | 0.0007890583903208837 |
| RochesterEFF_999_liver_4d | 0.0007890583903208837 |
| E2_1uM_embryo_4dpf | 0.0007797628603536453 |
| PKC412_40ugL_wholebody_6dpf | 0.0005962892461527877 |
| TB_500ngL_Ovary_192h | 0.0005917937927406628 |
| cypermethrin_1ugL_larvae_48hr | 0.0005817569058556842 |
| BDE_12500ngG_embryo_8days | 0.0005504208426025733 |Figure S1
