# Supplementary material for: Fish connectivity mapping: linking chemical stressors by their mechanisms of action-driven transcriptomic profiles
Source: BMC Genomics. 2016 Jan 28;17:84. doi: 10.1186/s12864-016-2406-y (PMC4730593; doi:10.1186/s12864-016-2406-y)
Supplement: Additional file 4: Table S3. — Cmap across ZF 21K and ZF 43K with the maximum signature size of 100 probes. (DOCX 14 kb) [file 12864_2016_2406_MOESM4_ESM.docx]

Table S3. Top five significant hits, if any, of selected Cmap queries across platforms within zebrafish (target IDs + source LogFC). ROGL hits are ranked by their adjusted connectivity scores and filtered by a p-value cutoff of 1/(number of sets of ROGLs). Informative connections, defined as a signature and one of its top five ROGL hits sharing the same or similar class of chemicals, are highlighted in bold.

| Signatures | 1^st^ match | 2^nd^ match | 3^rd^ match | 4^th^ match | 5^th^ match |
| --- | --- | --- | --- | --- | --- |
|  | From ZF 21K to ZF 43K (p-value 0.026; signature size average =49, min=15, max=81) | | | | |
| EE2_30ngL_Ovary_96hr.sig.EntrySwapped | PKC412_40ugL_wholebody | **GEN_2.2mgL_embryo** | **E2_1uM_embryo** | LIN_1.3mgL_embryo | PKC412_2ugL_wholebody |
| EE2_30ngL_M_Brain_48hr.sig.EntrySwapped | LIN_1.3mgL_embryo | LIN_1.2mgL_embryo | PKC412_40ugL_wholebody | PKC412_2ugL_wholebody | BPA_10ugL_ovary |
| EE2_30ngL_M_Brain_48hr.sig.EntrySwapped | LIN_1.3mgL_embryo | PKC412_2ugL_wholebody | LIN_1.2mgL_embryo |  |  |
| EE2_30ngL_M_Brain_96hr.sig.EntrySwapped | FLU_1700ugL_Ovary | LIN_1.3mgL_embryo | LIN_1.2mgL_embryo |  |  |
| EE2_30ngL_M_Liver_48hr.sig.EntrySwapped | **E2_5ugL_M_Liver** | LIN_1.3mgL_embryo | PKC412_40ugL_wholebody | LIN_1.2mgL_embryo | FLU_1.2mgL_embryo |
| EE2_30ngL_M_Testis_48hr.sig.EntrySwapped | **E2_1uM_embryo** |  |  |  |  |
| EE2_30ngL_M_Testis_48hr.sig.EntrySwapped | LIN_1.3mgL_embryo | LIN_1.2mgL_embryo | PRO_500ugL_F_Brain | BPA_10ugL_ovary | PKC412_40ugL_wholebody |
| FLU_1700ugL_Ovary_48hr.sig.EntrySwapped | PKC412_40ugL_wholebody | **FLU_1.2mgL_embryo** | BPA_10ugL_ovary | RDX_0.9mgL_fry |  |
| FLU_1700ugL_M_Testis_24hr.sig.EntrySwapped | RDX_15mgL_fry | **FLU_1700ugL_Ovary** | LIN_1.2mgL_embryo |  |  |
| FLU_1700ugL_M_Testis_48hr.sig.EntrySwapped | LIN_1.2mgL_embryo | LIN_1.3mgL_embryo | E2_5ugL_M_Liver | **FLU_1700ugL_Ovary** | DIA_273ugL_brain |
| PRO_500ugL_F_Brain_48hr.sig.EntrySwapped | FLU_1.2mgL_embryo | E2_5ugL_M_Liver |  |  |  |
| PRO_500ugL_Ovary_48hr.sig.EntrySwapped | BPA_8mgL_embryo |  |  |  |  |
| PRO_500ugL_M_Testis_48hr.sig.EntrySwapped | E2_5ugL_M_Liver |  |  |  |  |
|  | From ZF 43K to ZF 21K (p-value 0.022; signature size average = 37, min=5, max=65) | | | | |
| E2_1uM_embryo_4dpf.sig.EntrySwapped | **EE2_30ngL_M_Testis** | **EE2_30ngL_M_Liver** | TRB_3ugL_F_Liver | O2_3mgL_Testis | O2_1mgL_Ovary |
| GEN_2.4mgL_embryo_48hr.sig.EntrySwapped | DNP_14.2uM_embryo | DMB_509uM_embryo | TCDD_2nM_embryo | TRI_2500ugL_Ovary | TCDD_1ngmL_embryo |
| GEN_2.4mgL_embryo_48hr.sig.EntrySwapped | APM_5.83uM_embryo | TCDD_1ngmL_embryo | tBHQ_10uM_embryo |  |  |
| PRO_1.7mgL_embryo_48hr.sig.EntrySwapped | DMB_509uM_embryo | TRB_3ugL_F_Brain | tBHQ_10uM_embryo | TCDD_1ngmL_embryo | O2_1mgL_Testis |
| PRO_2mgL_embryo_48hr.sig.EntrySwapped | DMB_509uM_embryo | tBHQ_10uM_embryo | APM_5.83uM_embryo | **PRO_500ugL_F_Brain** | EE2_30ngL_M_Liver |
| E2_5ugL_M_Liver_12hrs.sig.EntrySwapped | TRB_3ugL_F_Liver | **EE2_30ngL_M_Liver** | O2_1mgL_Ovary | tBHQ_10uM_embryo | DNP_14.2uM_embryo |
| E2_5ugL_M_Liver_24hrs.sig.EntrySwapped | **EE2_30ngL_M_Liver** | TRB_3ugL_F_Liver | KET_370ugL_M_Liver | O2_3mgL_Testis |  |
| E2_5ugL_M_Liver_48hrs.sig.EntrySwapped | **EE2_30ngL_M_Liver** | TRB_3ugL_F_Liver | TRI_2500ugL_Ovary | KET_370ugL_M_Liver | TCDD_2nM_embryo |
| E2_5ugL_M_Liver_4hrs.sig.EntrySwapped | **EE2_30ngL_M_Liver** | TRB_3ugL_F_Liver | O2_1mgL_Testis | O2_3mgL_Testis | TCDD_2nM_embryo |
| FLU_1700ugL_Ovary_48hr.sig.EntrySwapped | MUS_500ugL_Ovary | EE2_30ngL_M_Liver | KET_370ugL_M_Brain | VIN_1000ugL_Ovary | TRB_3ugL_F_Liver |
| PRO_500ugL_F_Brain_48hr.sig.EntrySwapped | MUS_500ugL_Ovary | VIN_1000ugL_Ovary | MUS_500ugL_M_Brain | KET_370ugL_Ovary | FIP_5ugL_Ovary |
| PRO_500ugL_Ovary_48hr.sig.EntrySwapped | MUS_500ugL_Ovary | EE2_30ngL_M_Liver | TRB_3ugL_F_Liver | KET_370ugL_M_Brain | VIN_1000ugL_Ovary |
