# Supplementary figures and images for: Fish connectivity mapping: linking chemical stressors by their mechanisms of action-driven transcriptomic profiles
Source: BMC Genomics. 2016 Jan 28;17:84. doi: 10.1186/s12864-016-2406-y (PMC4730593; doi:10.1186/s12864-016-2406-y)

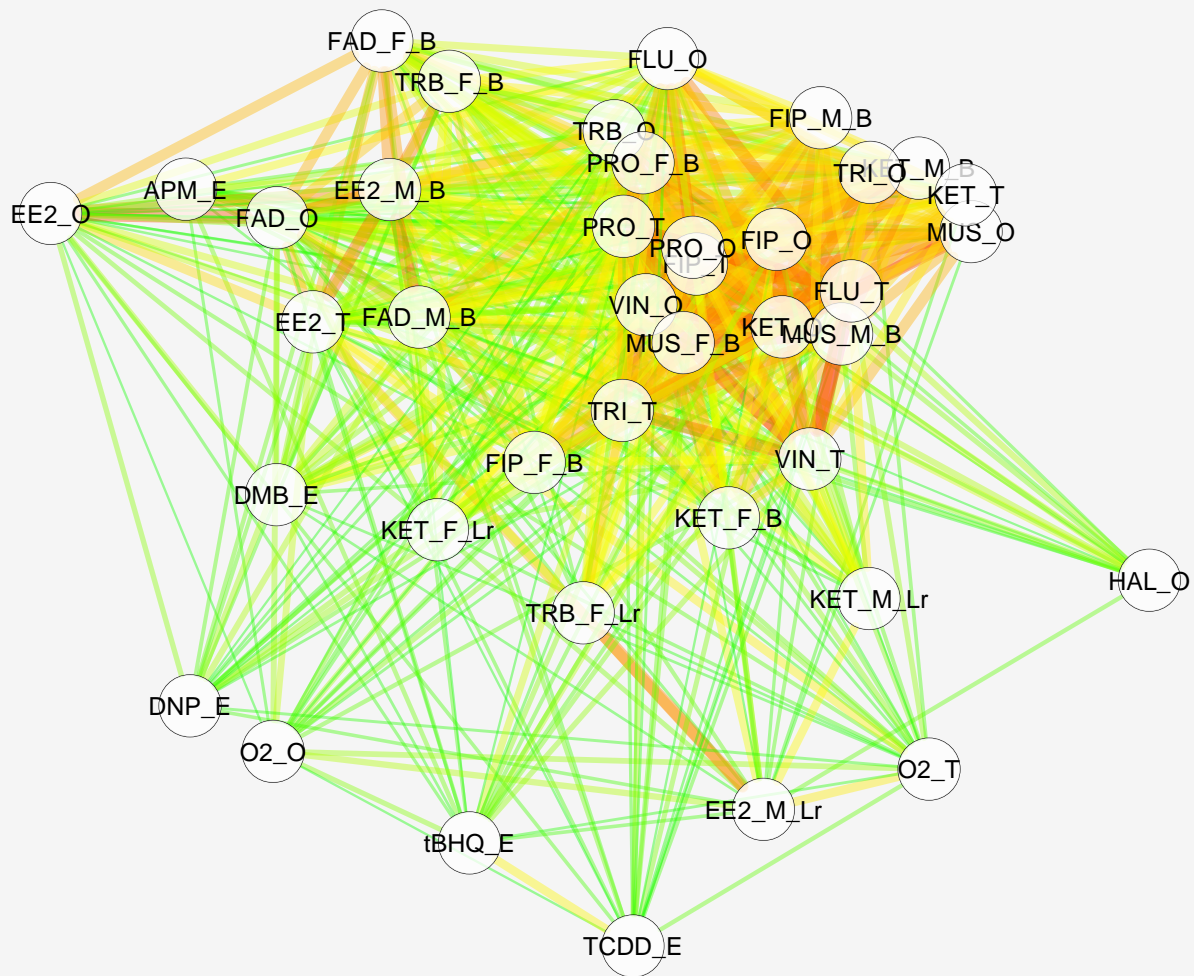

A

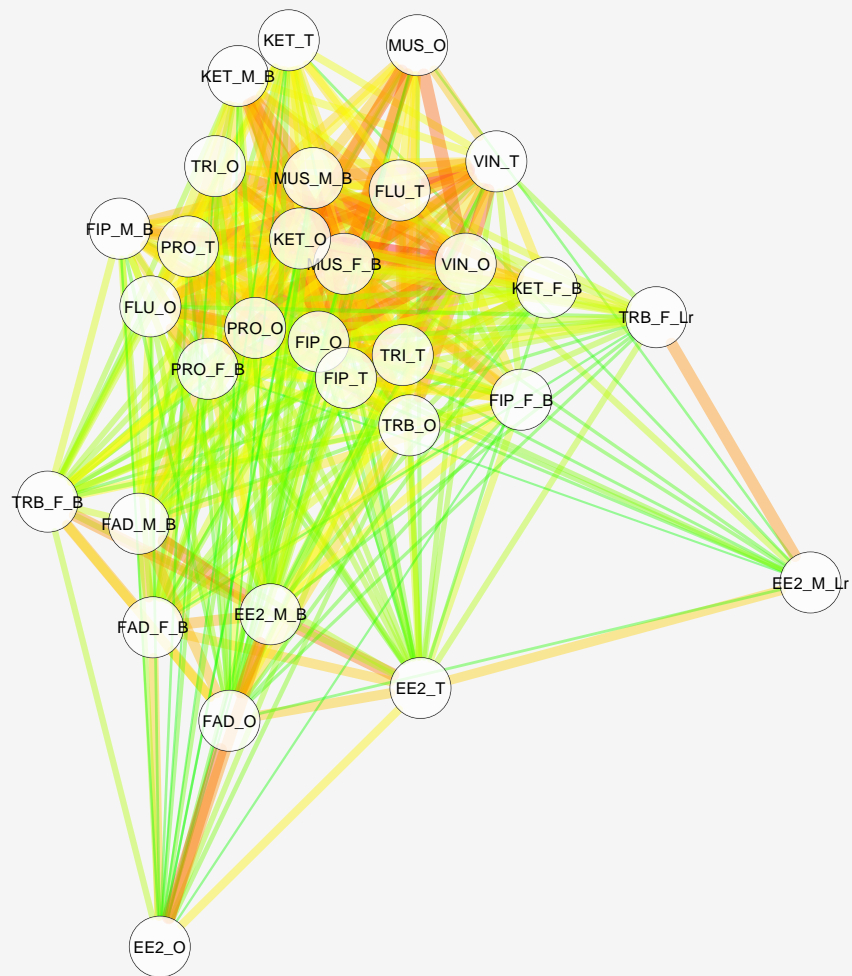

B

Supplement: Additional file 5: Figure S2. — A network view of chemicals and tissue types based on samples profiled on ZF 21K. Each chemical treatment condition and the tissue profiled is represented as a node. Two nodes are connected by an edge weighted by their connectivity score. A shorter, darker, and wider edge between two nodes denote a higher connectivity score. All connections shown are statistically significant. A) 40 nodes with 573 connections; B) 30 nodes with 380 connections with each node having a minimum connectivity score of 10 in at least one of its connections. Tissue types: T, testis; O, ovary; B, brain; Lr, liver. (PDF 37 kb) [file 12864_2016_2406_MOESM5_ESM.pdf]
