# Supplementary material for: Fish connectivity mapping: linking chemical stressors by their mechanisms of action-driven transcriptomic profiles
Source: BMC Genomics. 2016 Jan 28;17:84. doi: 10.1186/s12864-016-2406-y (PMC4730593; doi:10.1186/s12864-016-2406-y)
Supplement: Additional file 6: Figure S3. — Relationships between connectivity strength and DEGs. A total of 106 chemical conditions with relative transcriptomic impact ≥ 0.001 were considered. Connectivity scores were normalized to the size of respective query signatures. A) average LogFC vs connectivity scores; B) average logFC vs relative transcriptomic impact (RTI, percentage of transcriptome as DEGs); C) connectivity scores vs RTIs. (PPTX 49 kb) [file 12864_2016_2406_MOESM6_ESM.pptx]

## Slide 1
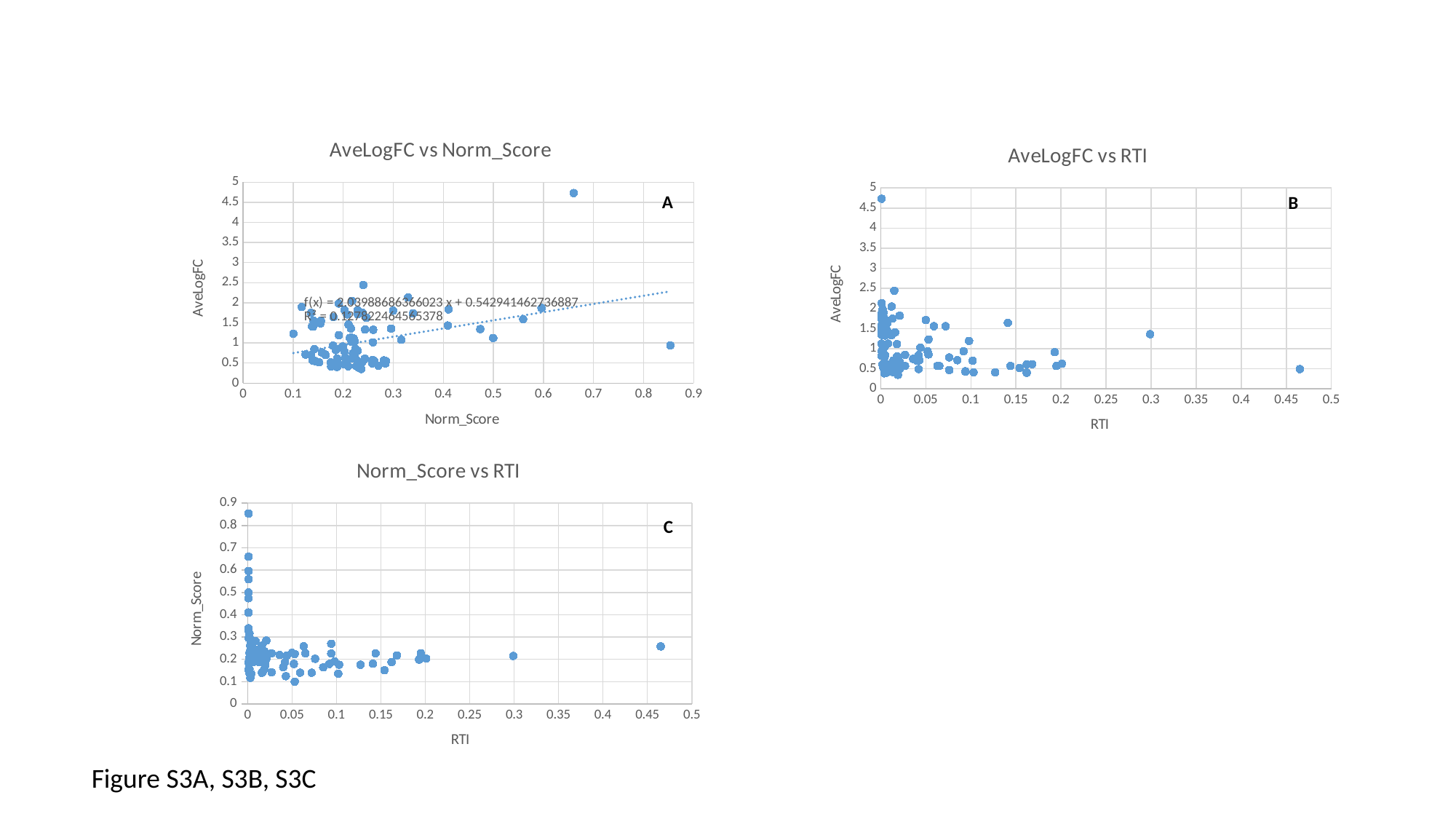

### Chart: AveLogFC vs Norm_Score
| Category | AveLogFC |
|---|---|
### Chart: AveLogFC vs RTI
| Category | AveLogFC |
|---|---|A
B
### Chart: Norm_Score vs RTI
| Category | Norm_Score |
|---|---|C
Figure S3A, S3B, S3C
